# Correction: The Genomic Signature of Crop-Wild Introgression in Maize

**DOI:** 10.1371/annotation/2eef7b5b-29b2-412f-8472-8fd7f9bd65ab

**Published:** 2013-09-19

**Authors:** Matthew B. Hufford, Pesach Lubinksy, Tanja Pyhäjärvi, Michael T. Devengenzo, Norman C. Ellstrand, Jeffrey Ross-Ibarra

In the list of authors, the second author's name is incorrectly spelled. The correct spelling is Pesach Lubinsky.

