# Supplementary figures and images for: The Genomic Signature of Crop-Wild Introgression in Maize
Source: PLoS Genet. 2013 May 9;9(5):e1003477. doi: 10.1371/journal.pgen.1003477 (PMC3649989; doi:10.1371/journal.pgen.1003477)

← *mexicana* → ← maize → ← references →

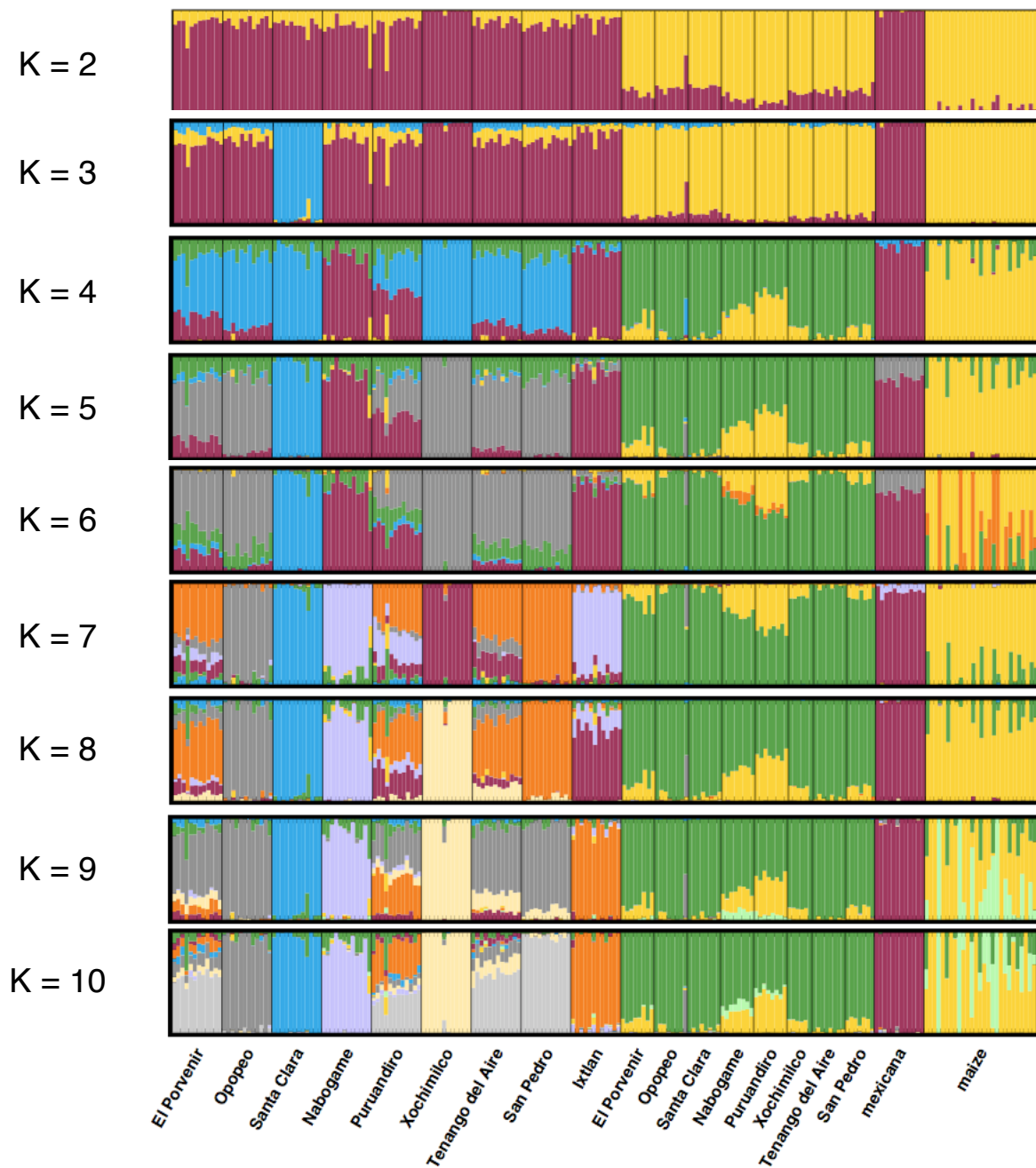

Supplement: Figure S1 — Bar plot of assignment proportions from STRUCTURE analysis at K = 2-K = 10 for mexicana and maize individuals. The Ixtlan maize population was excluded from this figure and the STRUCTURE analysis. (PDF) [file pgen.1003477.s001.pdf]

## Genome-wide Distribution of f3 Statistic

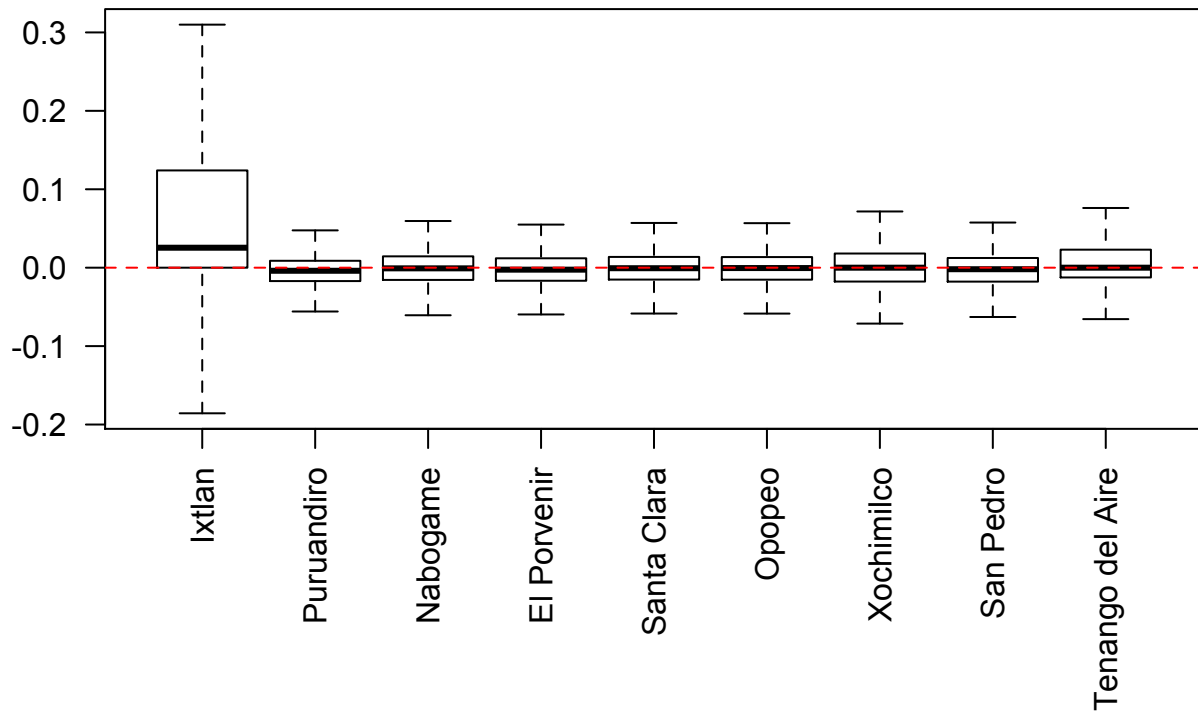

Supplement: Figure S2 — Distribution of f3 statistic in genome-wide SNPs genotyped in nine maize populations that are sympatric with mexicana. (PDF) [file pgen.1003477.s002.pdf]

A

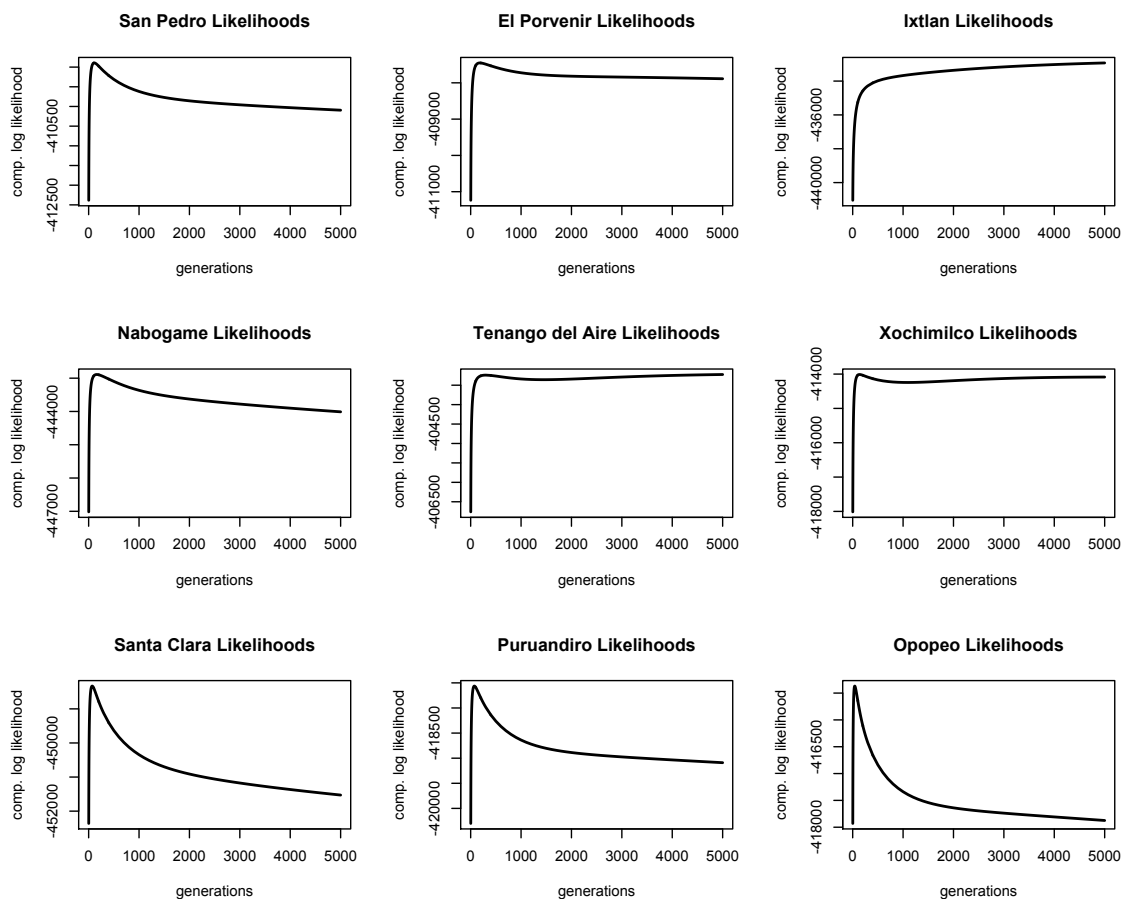

B

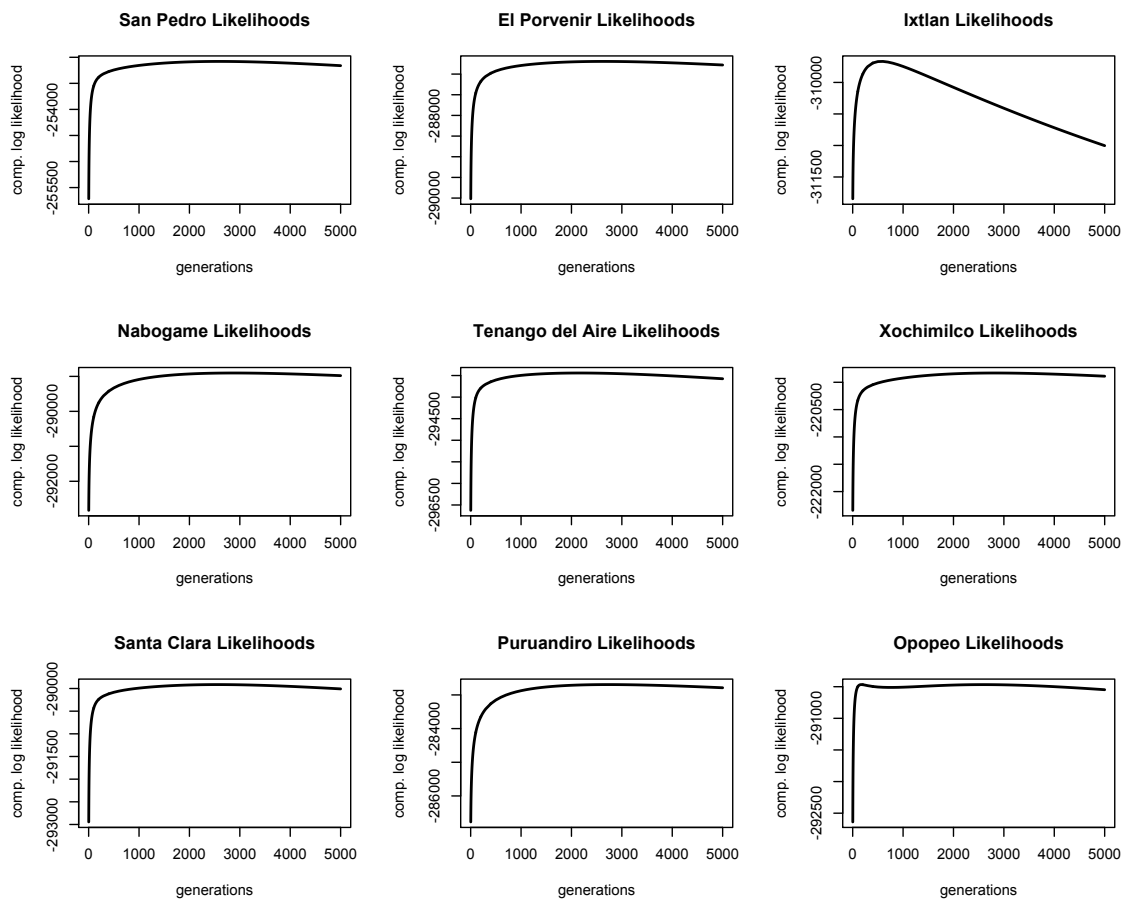

Supplement: Figure S4 — Likelihood plots across generations since admixture for each population for both mexicana (A) and maize (B). (PDF) [file pgen.1003477.s004.pdf]

A

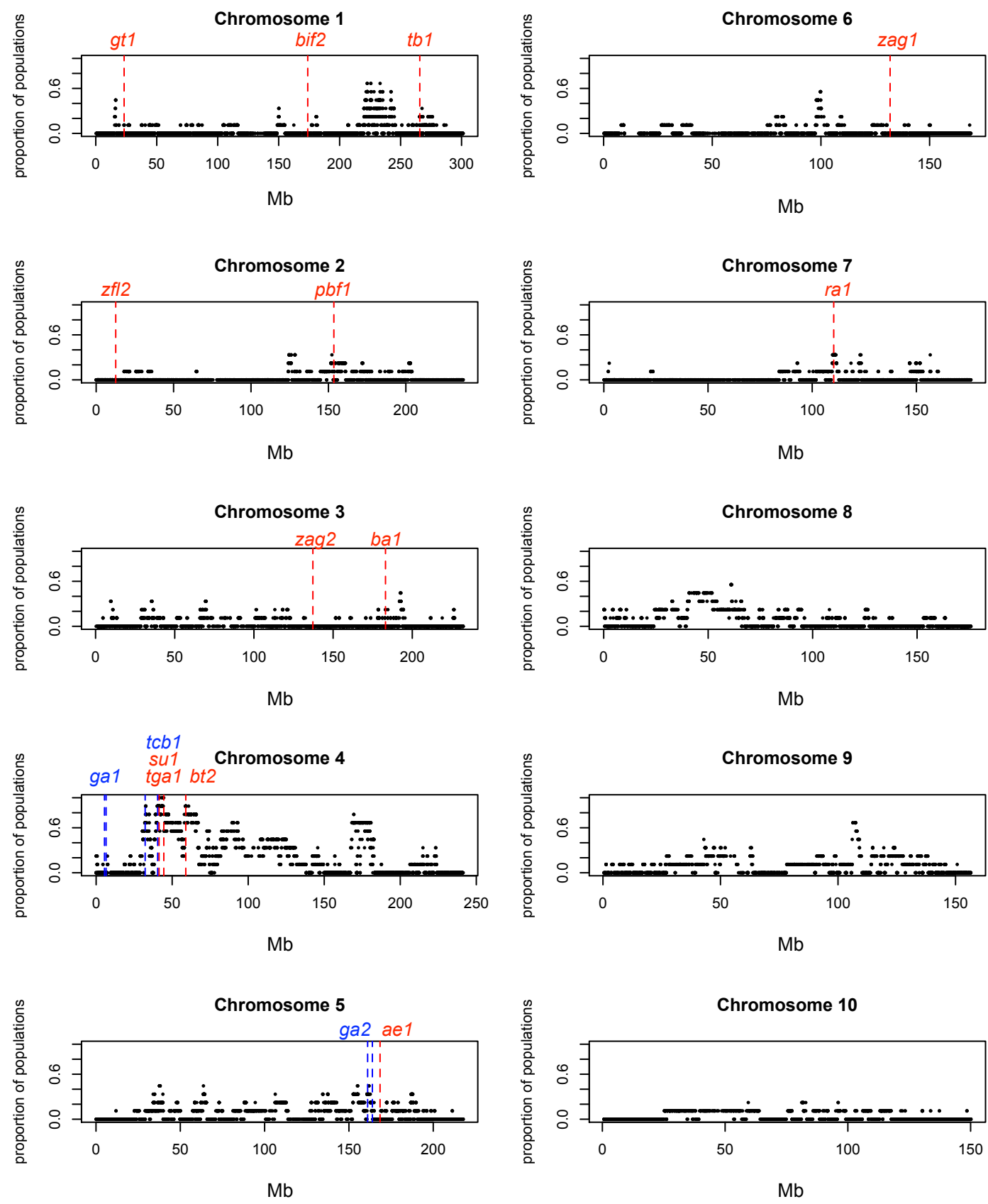

B

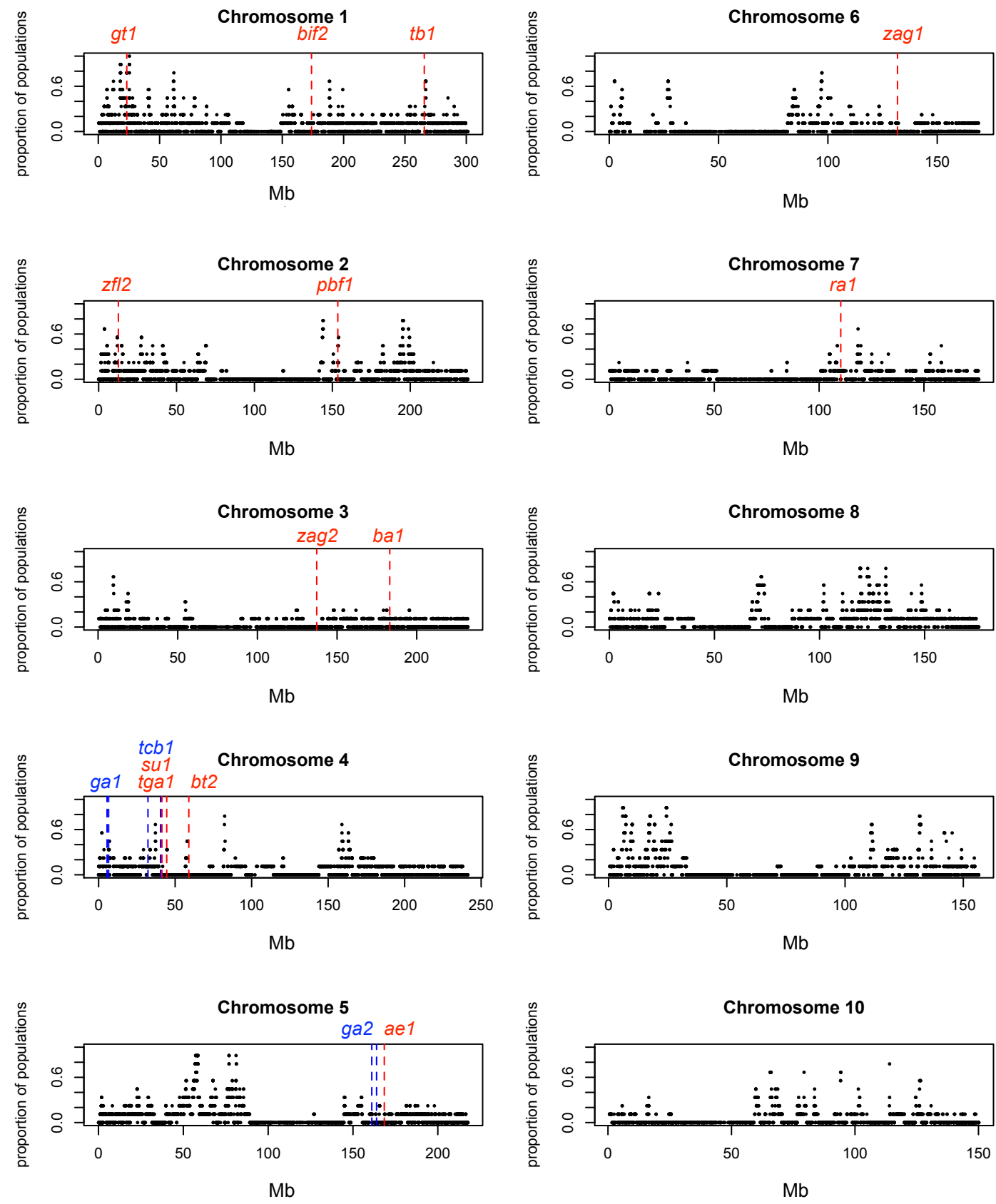

Supplement: Figure S5 — Proportion of populations showing resistance to introgression across each chromosome for maize-to-mexicana (A) and mexicana-to-maize (B) introgression. Thirteen well-known domestication loci (red) and three characterized pollen-pistil cross-incompatibility loci (blue) are indicated with dashed lines and labeled above the plots. (PDF) [file pgen.1003477.s005.pdf]

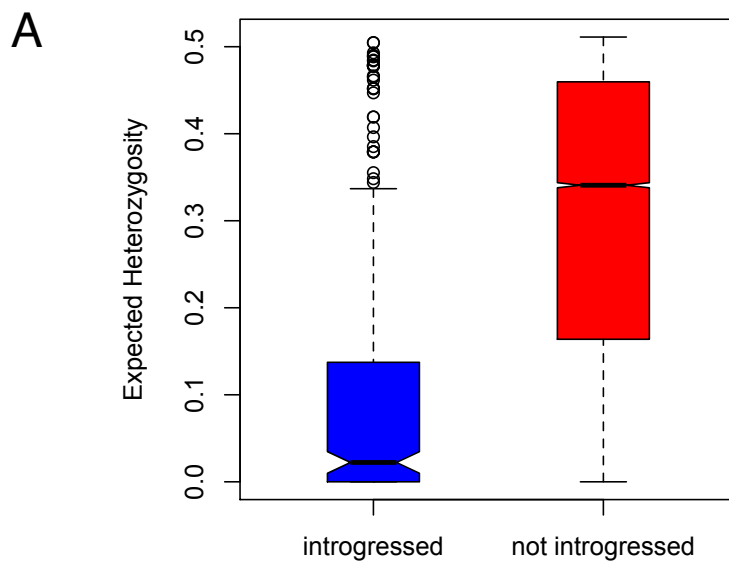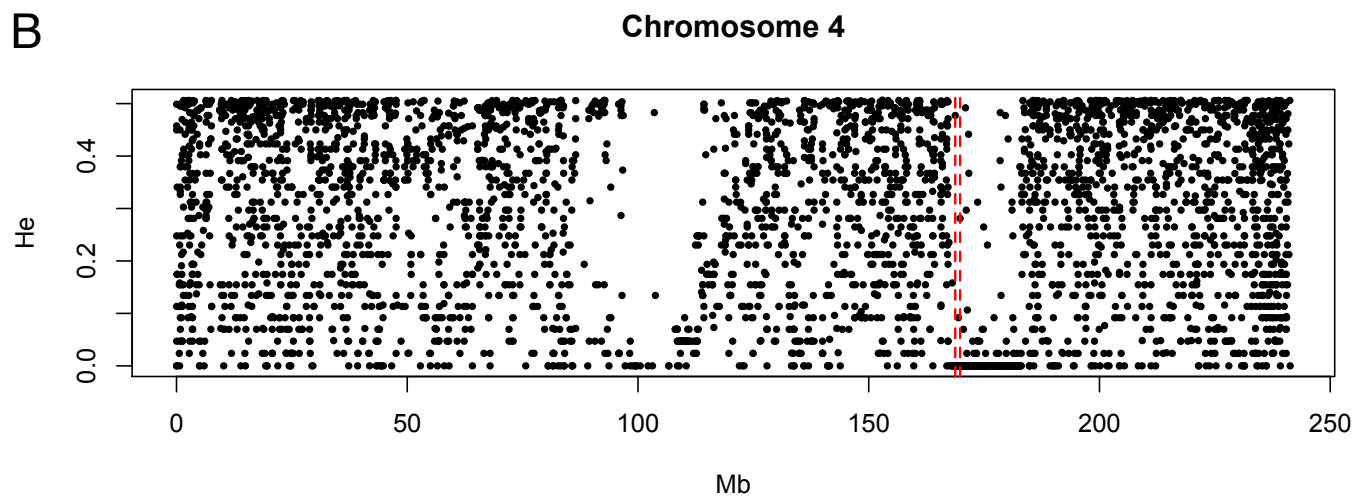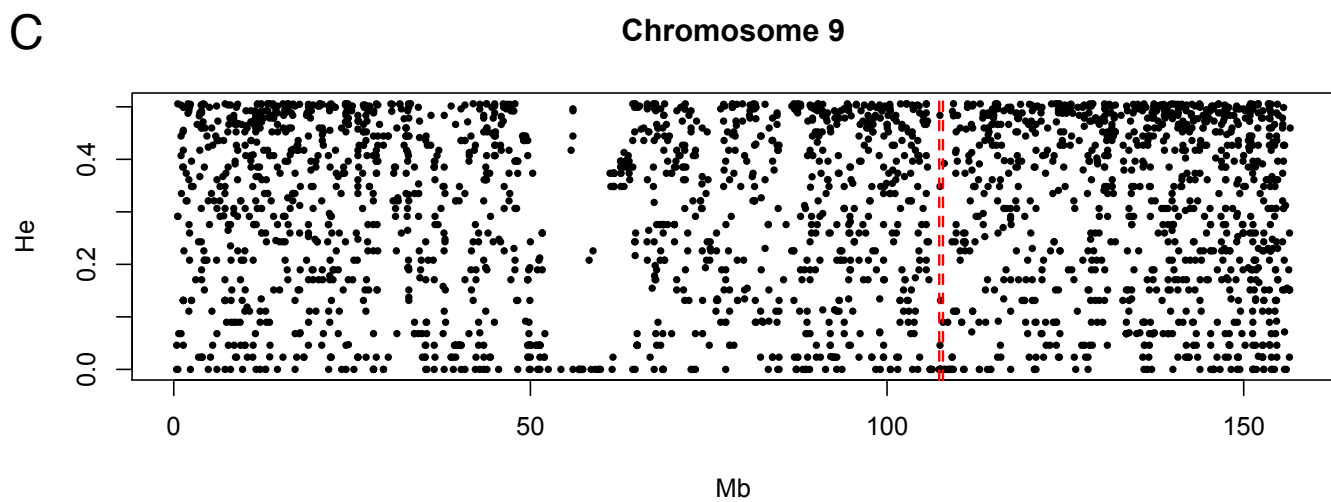

Supplement: Figure S6 — Evidence supporting reduced haplotype diversity in shared regions of mexicana-to-maize introgression. (A) Expected heterozygosity in introgressed (blue) and non-introgressed (red) individuals in shared regions. Expected heterozygosity plotted across chromosomes 4 (B) and 9 (C) in introgressed individuals. Regions of introgression shared across populations are indicated by a red dashed line. (PDF) [file pgen.1003477.s006.pdf]

## Plant Height at 30 Days

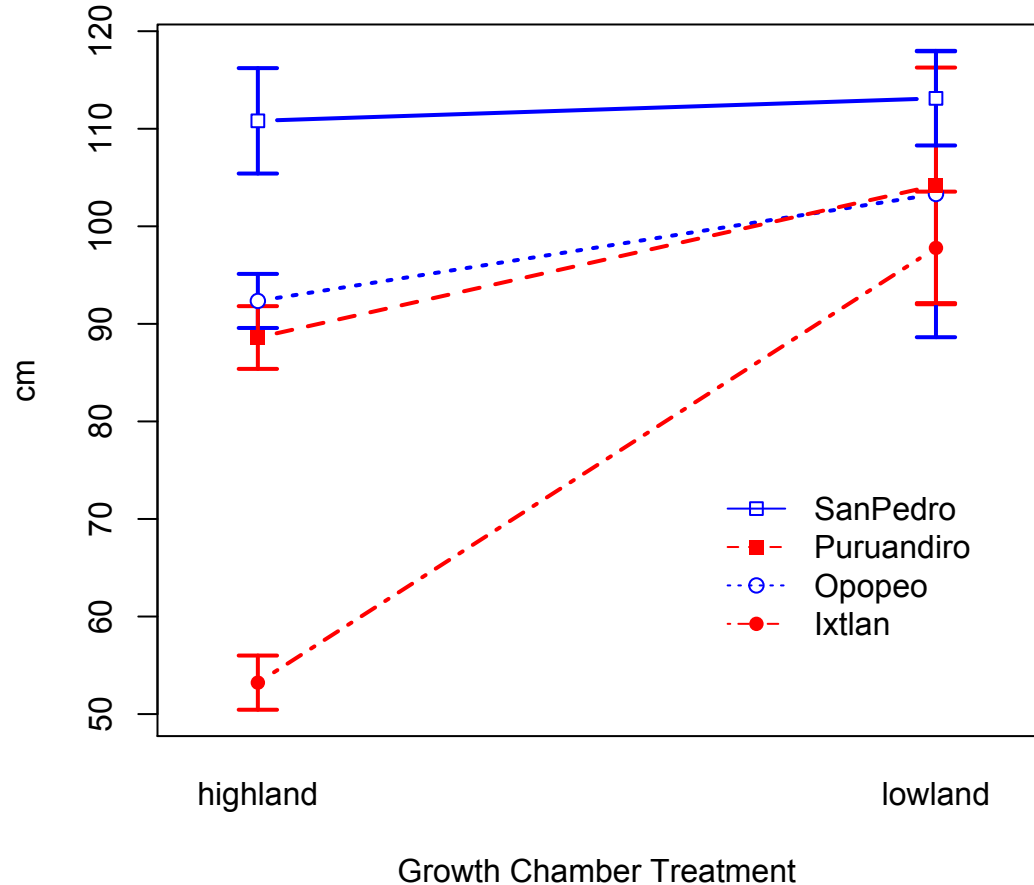

Supplement: Figure S7 — Plant height at 30 days in maize populations with (blue) and without (red) introgression at loci depicted in Figure 4 under highland and lowland conditions. Confidence interval is +/−1 standard error. (PDF) [file pgen.1003477.s007.pdf]
