# Supplementary material for: The Genomic Signature of Crop-Wild Introgression in Maize
Source: PLoS Genet. 2013 May 9;9(5):e1003477. doi: 10.1371/journal.pgen.1003477 (PMC3649989; doi:10.1371/journal.pgen.1003477)
Supplement: Figure S3 — HAPMIX and STRUCTURE plots of introgression for each chromosome. Colors and axes are as in Figure 3. (PDF) [file pgen.1003477.s003.pdf]

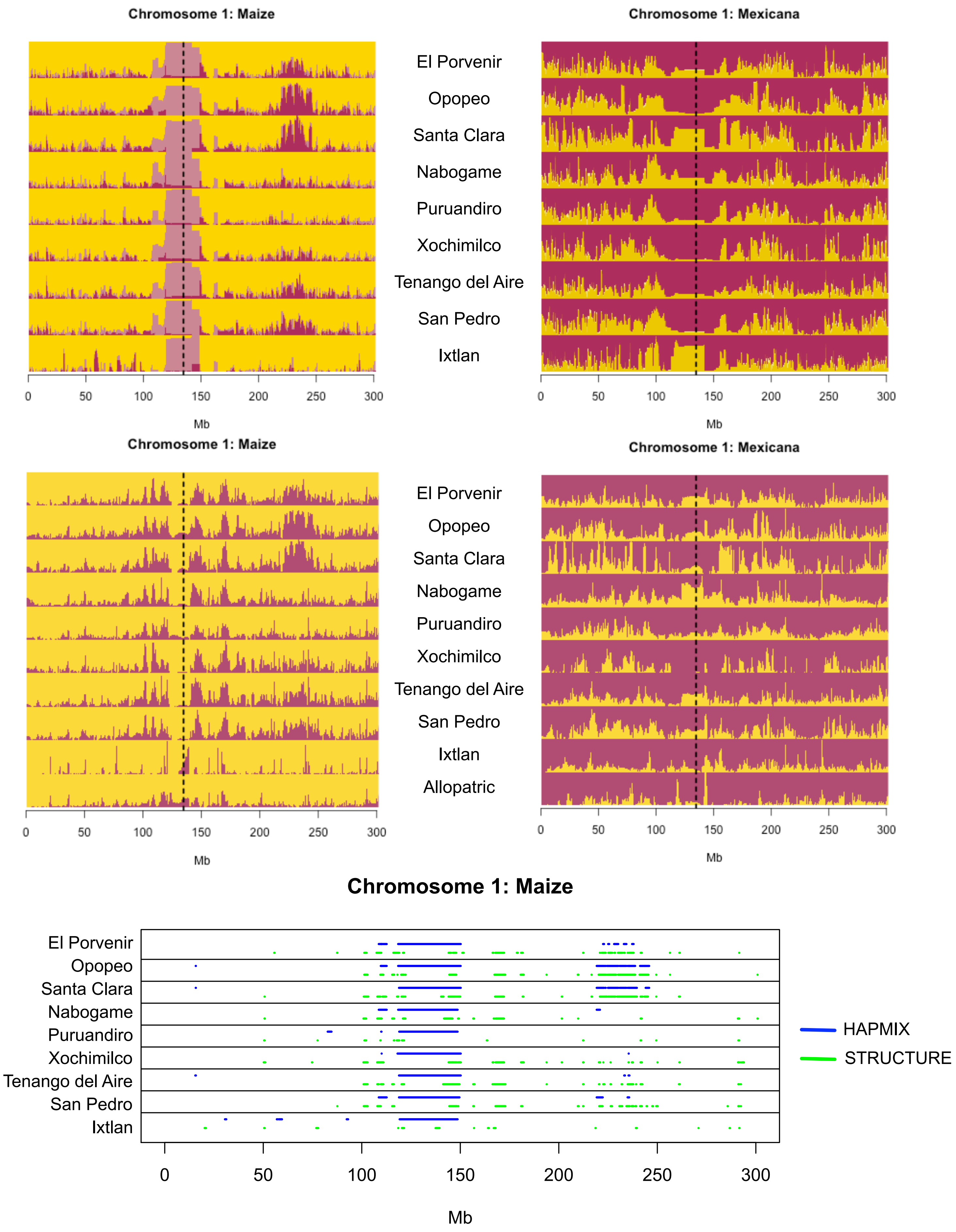
Chromosome 1: Maize
Chromosome 1: Mexicana
El Porvenir
Opopeo
Santa Clara
Nabogame
Puruandiro
Xochimilco
Tenango del Aire
San Pedro
Ixtlan
0 50 100 150 200 250 300
Mb
Chromosome 1: Maize
Chromosome 1: Mexicana
El Porvenir
Opopeo
Santa Clara
Nabogame
Puruandiro
Xochimilco
Tenango del Aire
San Pedro
Ixtlan
Allopatric
0 50 100 150 200 250 300
Mb
Chromosome 1: Maize
El Porvenir
Opopeo
Santa Clara
Nabogame
Puruandiro
Xochimilco
Tenango del Aire
San Pedro
Ixtlan
HAPMIX
STRUCTURE
0 50 100 150 200 250 300
Mb

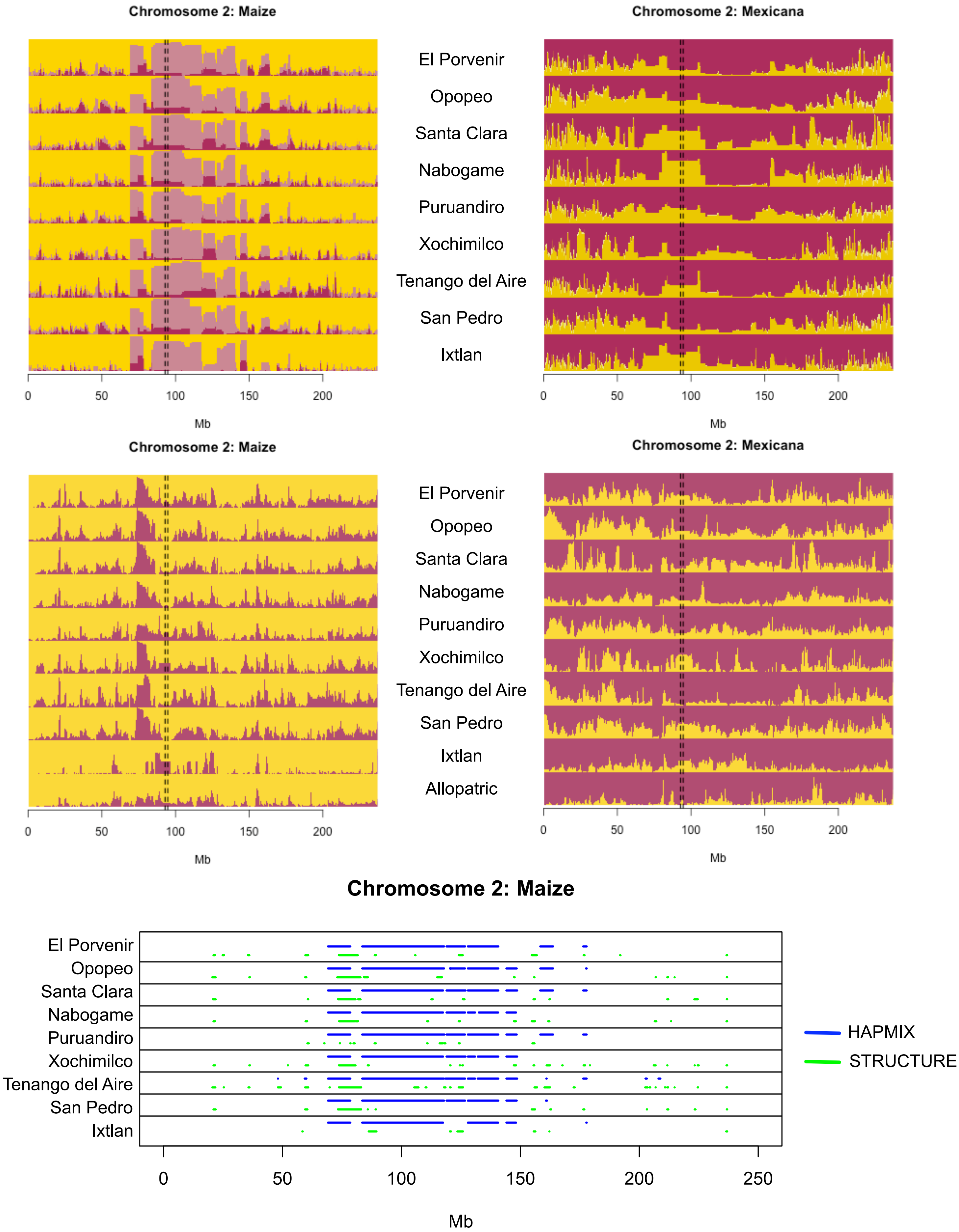

Chromosome 2: Maize
Chromosome 2: Mexicana
El Porvenir
Opopeo
Santa Clara
Nabogame
Puruandiro
Xochimilco
Tenango del Aire
San Pedro
Ixtlan
0
50
100
150
200
Mb
Chromosome 2: Maize
Chromosome 2: Mexicana
El Porvenir
Opopeo
Santa Clara
Nabogame
Puruandiro
Xochimilco
Tenango del Aire
San Pedro
Ixtlan
Allopatric
0
50
100
150
200
Mb
Chromosome 2: Maize
El Porvenir
Opopeo
Santa Clara
Nabogame
Puruandiro
Xochimilco
Tenango del Aire
San Pedro
Ixtlan
HAPMIX
STRUCTURE
0
50
100
150
200
250
Mb

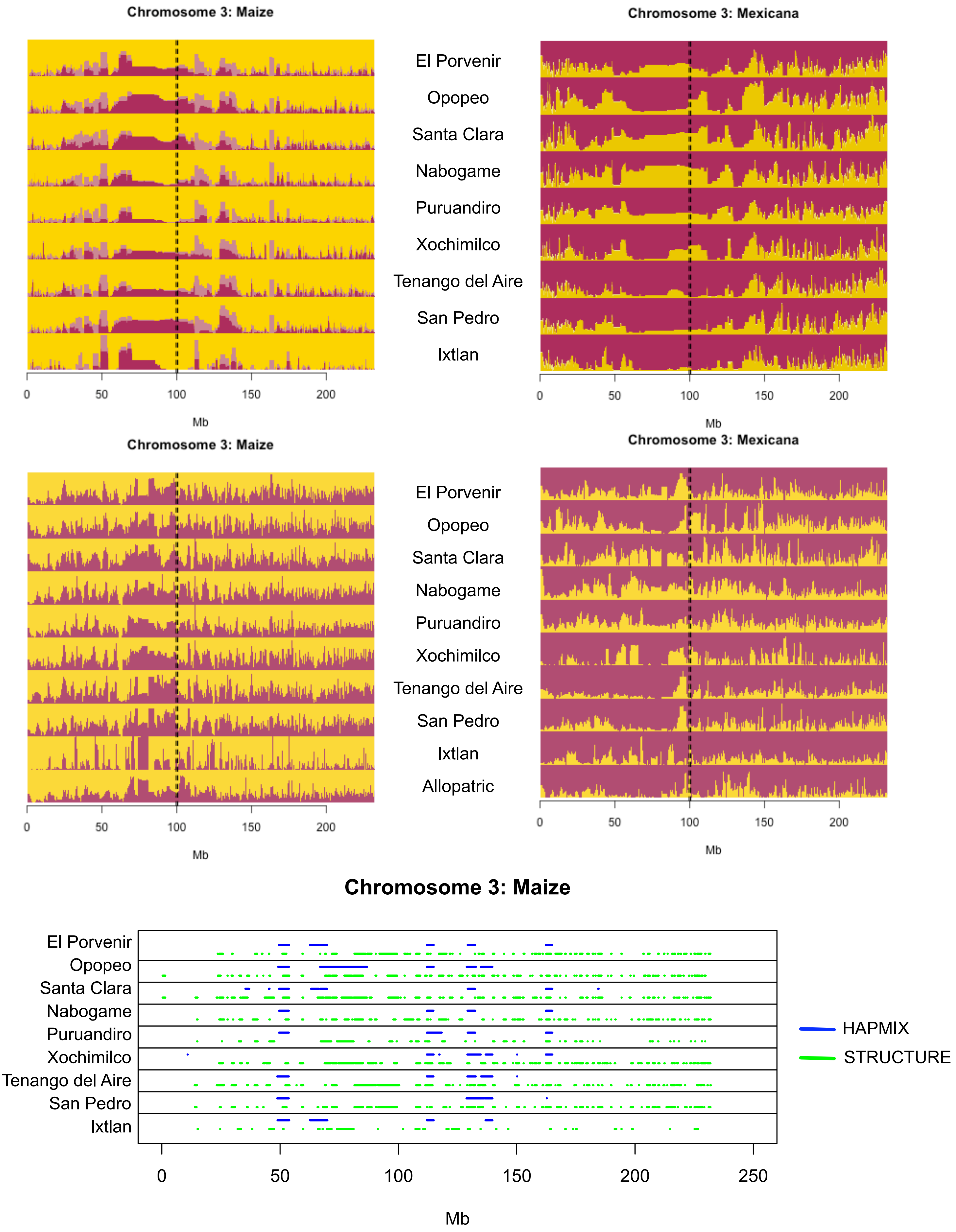

Chromosome 3: Maize
Chromosome 3: Mexicana
El Porvenir
Opopeo
Santa Clara
Nabogame
Puruandiro
Xochimilco
Tenango del Aire
San Pedro
Ixtlan
0
50
100
150
200
Mb
Chromosome 3: Maize
Chromosome 3: Mexicana
El Porvenir
Opopeo
Santa Clara
Nabogame
Puruandiro
Xochimilco
Tenango del Aire
San Pedro
Ixtlan
Allopatric
0
50
100
150
200
Mb
Chromosome 3: Maize
El Porvenir
Opopeo
Santa Clara
Nabogame
Puruandiro
Xochimilco
Tenango del Aire
San Pedro
Ixtlan
HAPMIX
STRUCTURE
0
50
100
150
200
250
Mb

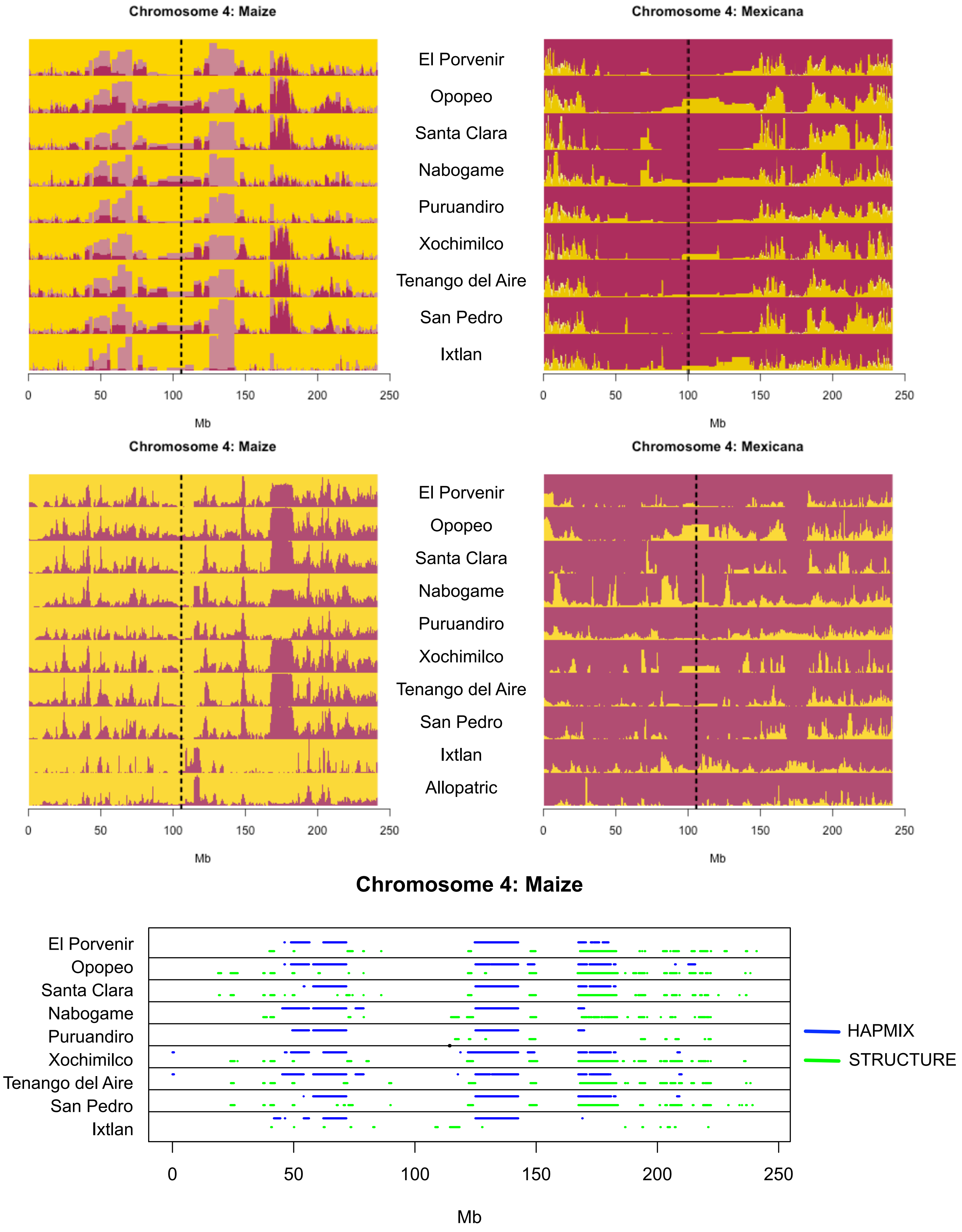
Chromosome 4: Maize
Chromosome 4: Mexicana
El Porvenir
Opopeo
Santa Clara
Nabogame
Puruandiro
Xochimilco
Tenango del Aire
San Pedro
Ixtlan
Allopatric
0
50
100
150
200
250
Mb
HAPMIX
STRUCTURE

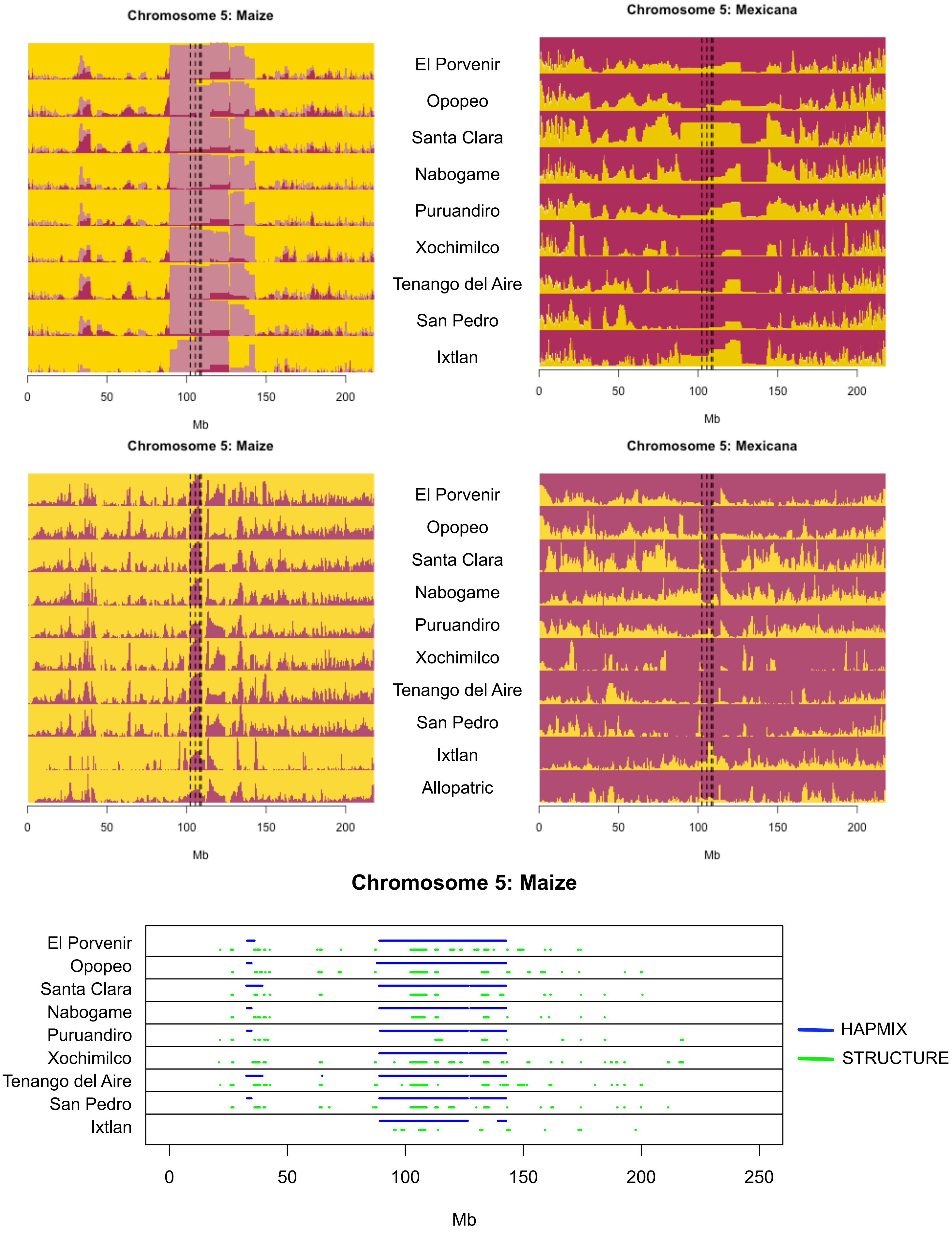
Chromosome 5: Maize
Chromosome 5: Mexicana
El Porvenir
Opopeo
Santa Clara
Nabogame
Puruandiro
Xochimilco
Tenango del Aire
San Pedro
Ixtlan
0 50 100 150 200
Mb
Chromosome 5: Maize
Chromosome 5: Mexicana
El Porvenir
Opopeo
Santa Clara
Nabogame
Puruandiro
Xochimilco
Tenango del Aire
San Pedro
Ixtlan
Allopatric
0 50 100 150 200
Mb
Chromosome 5: Maize
El Porvenir
Opopeo
Santa Clara
Nabogame
Puruandiro
Xochimilco
Tenango del Aire
San Pedro
Ixtlan
HAPMIX
STRUCTURE
0 50 100 150 200 250
Mb

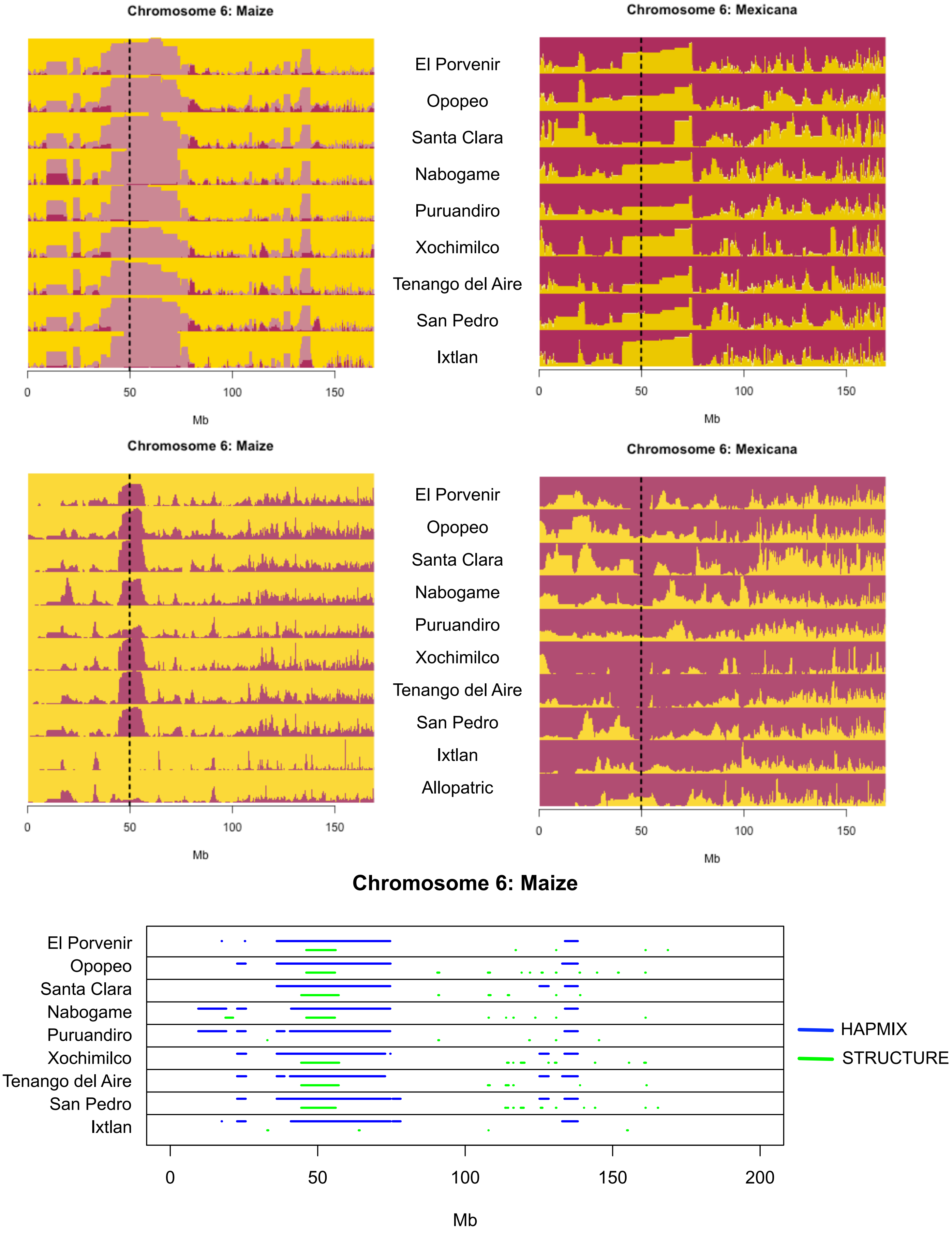
Chromosome 6: Maize
Chromosome 6: Mexicana
El Porvenir
Opopeo
Santa Clara
Nabogame
Puruandiro
Xochimilco
Tenango del Aire
San Pedro
Ixtlan
0
50
100
150
Mb
Chromosome 6: Maize
Chromosome 6: Mexicana
El Porvenir
Opopeo
Santa Clara
Nabogame
Puruandiro
Xochimilco
Tenango del Aire
San Pedro
Ixtlan
Allopatric
0
50
100
150
Mb
Chromosome 6: Maize
El Porvenir
Opopeo
Santa Clara
Nabogame
Puruandiro
Xochimilco
Tenango del Aire
San Pedro
Ixtlan
HAPMIX
STRUCTURE
0
50
100
150
200
Mb

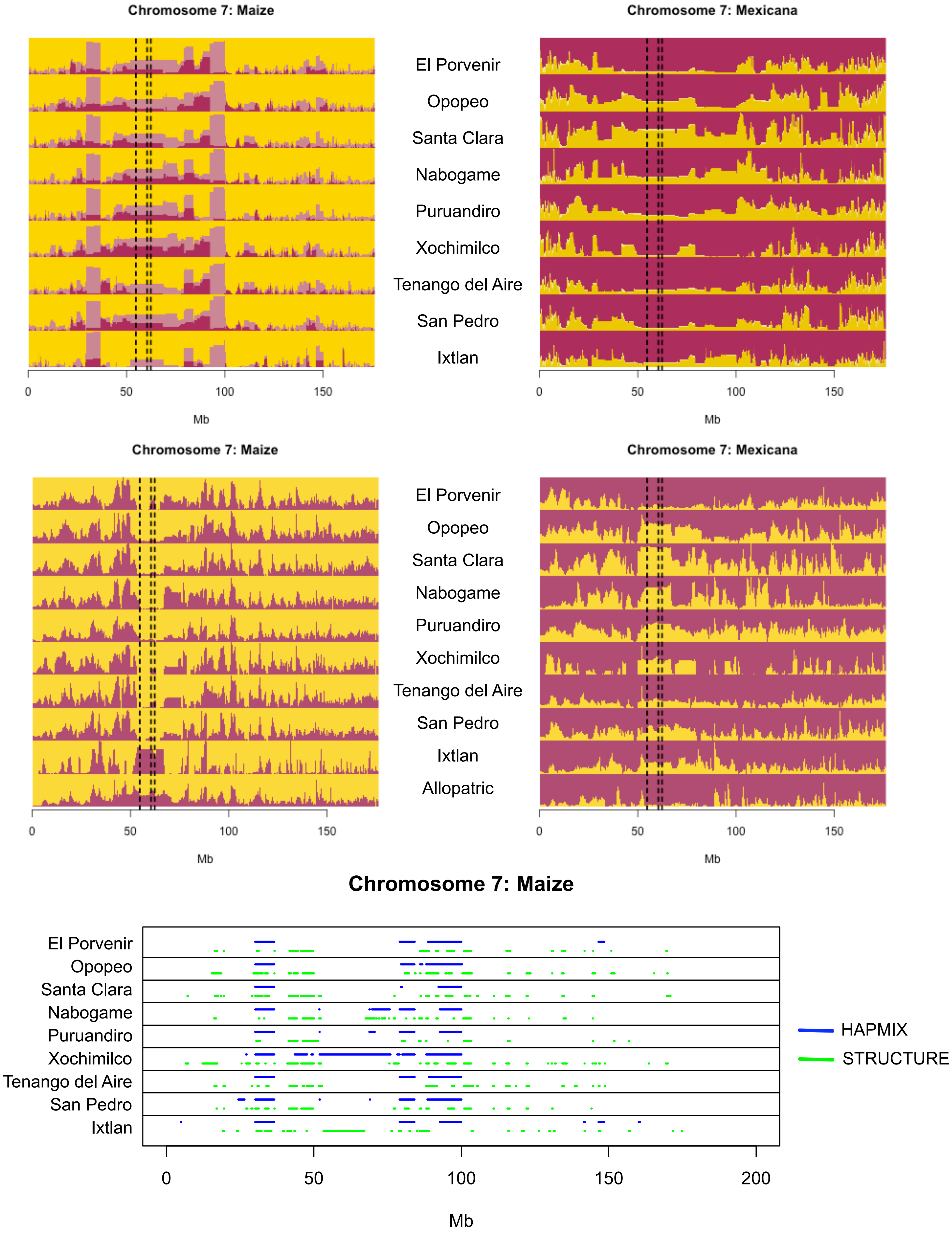

Chromosome 7: Maize
Chromosome 7: Mexicana
El Porvenir
Opopeo
Santa Clara
Nabogame
Puruandiro
Xochimilco
Tenango del Aire
San Pedro
Ixtlan
0
50
100
150
Mb
Chromosome 7: Maize
Chromosome 7: Mexicana
El Porvenir
Opopeo
Santa Clara
Nabogame
Puruandiro
Xochimilco
Tenango del Aire
San Pedro
Ixtlan
Allopatric
0
50
100
150
Mb
Chromosome 7: Maize
El Porvenir
Opopeo
Santa Clara
Nabogame
Puruandiro
Xochimilco
Tenango del Aire
San Pedro
Ixtlan
HAPMIX
STRUCTURE
0
50
100
150
200
Mb

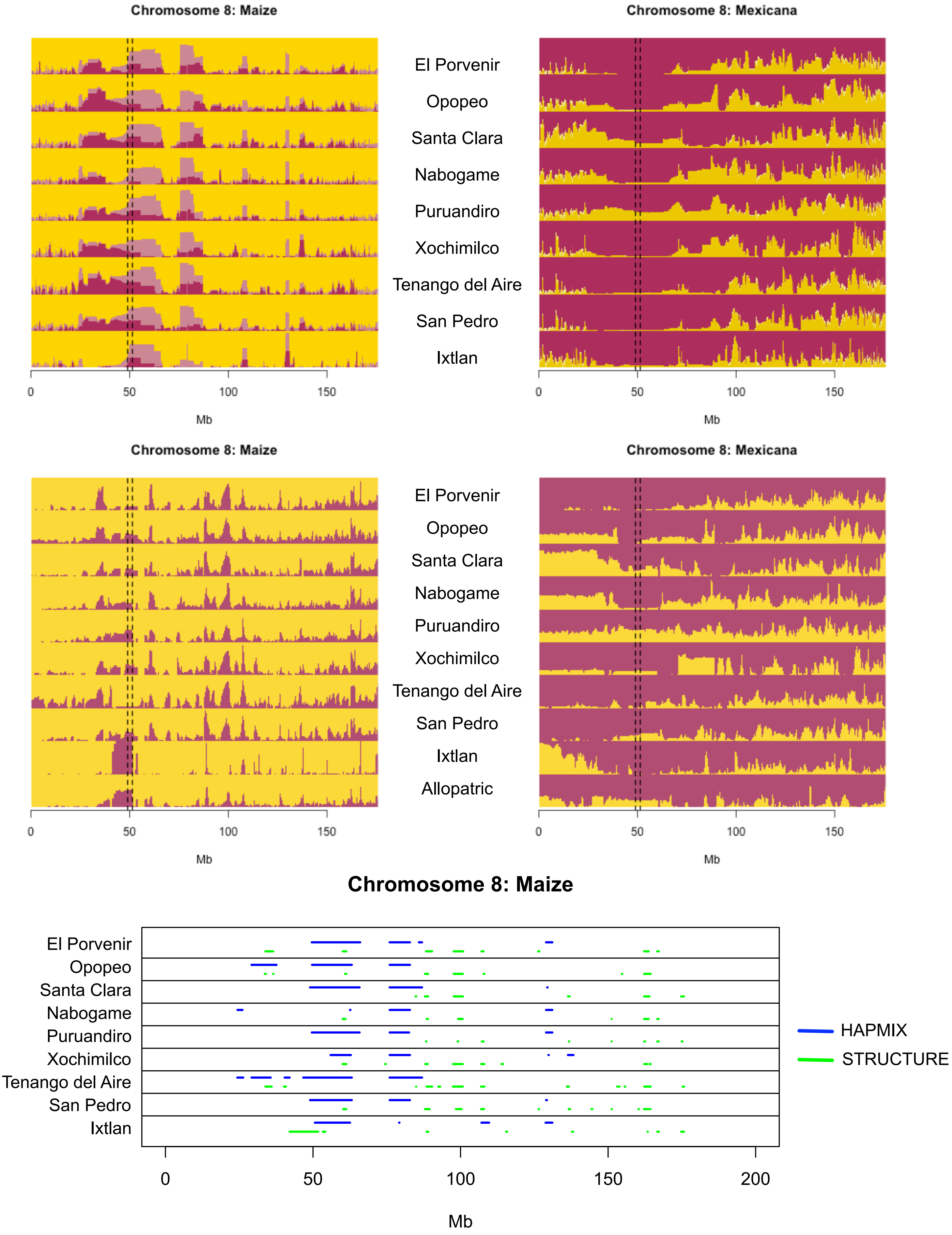

Chromosome 8: Maize
Chromosome 8: Mexicana
El Porvenir
Opopeo
Santa Clara
Nabogame
Puruandiro
Xochimilco
Tenango del Aire
San Pedro
Ixtlan
0
50
100
150
Mb
Chromosome 8: Maize
Chromosome 8: Mexicana
El Porvenir
Opopeo
Santa Clara
Nabogame
Puruandiro
Xochimilco
Tenango del Aire
San Pedro
Ixtlan
Allopatric
0
50
100
150
Mb
Chromosome 8: Maize
El Porvenir
Opopeo
Santa Clara
Nabogame
Puruandiro
Xochimilco
Tenango del Aire
San Pedro
Ixtlan
HAPMIX
STRUCTURE
0
50
100
150
200
Mb

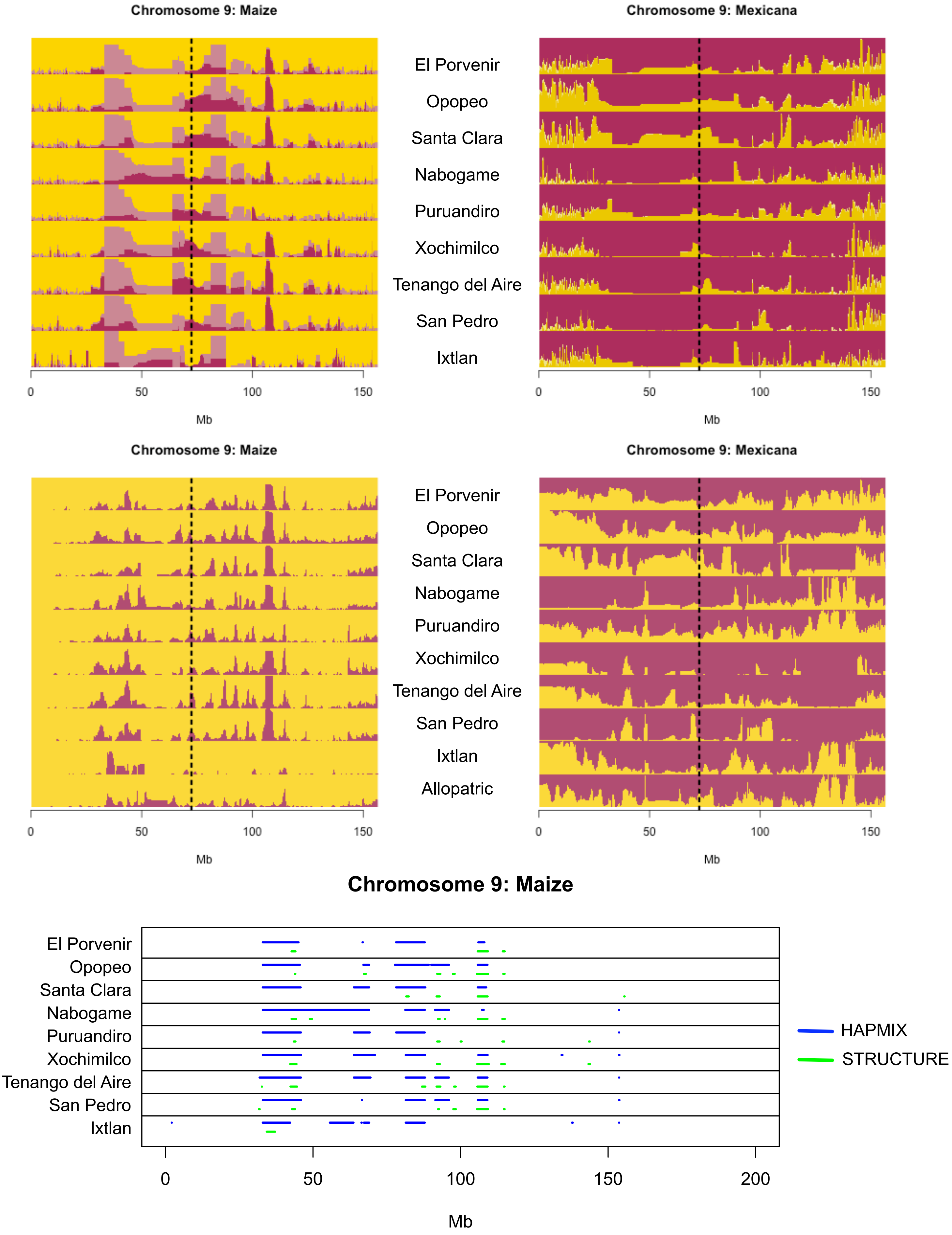

Chromosome 9: Maize
Chromosome 9: Mexicana
El Porvenir
Opopeo
Santa Clara
Nabogame
Puruandiro
Xochimilco
Tenango del Aire
San Pedro
Ixtlan
0
50
100
150
Mb
Chromosome 9: Maize
Chromosome 9: Mexicana
El Porvenir
Opopeo
Santa Clara
Nabogame
Puruandiro
Xochimilco
Tenango del Aire
San Pedro
Ixtlan
Allopatric
0
50
100
150
Mb
Chromosome 9: Maize
El Porvenir
Opopeo
Santa Clara
Nabogame
Puruandiro
Xochimilco
Tenango del Aire
San Pedro
Ixtlan
HAPMIX
STRUCTURE
0
50
100
150
200
Mb

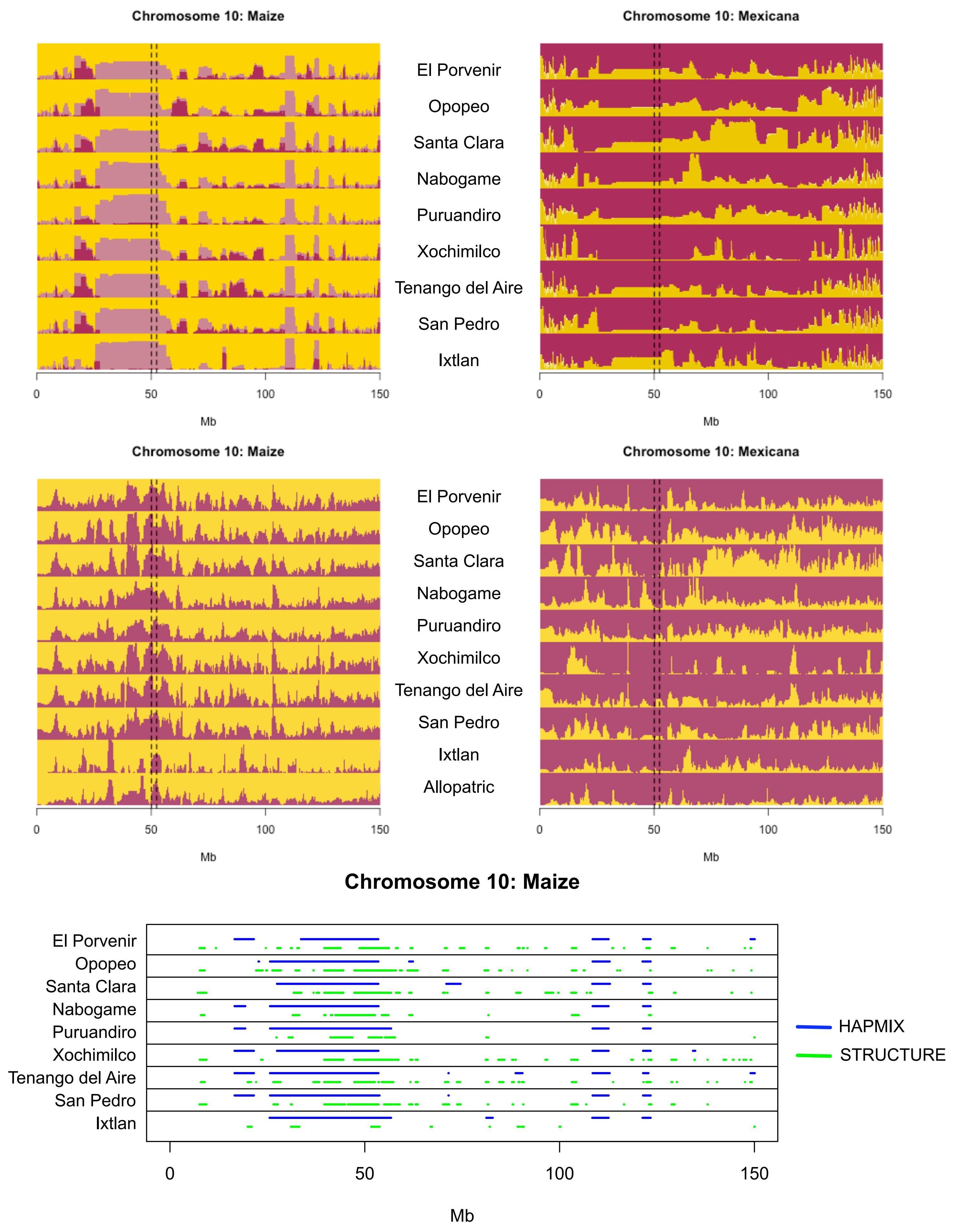
Chromosome 10: Maize
Chromosome 10: Mexicana
El Porvenir
Opopeo
Santa Clara
Nabogame
Puruandiro
Xochimilco
Tenango del Aire
San Pedro
Ixtlan
0
50
100
150
Mb
Chromosome 10: Maize
Chromosome 10: Mexicana
El Porvenir
Opopeo
Santa Clara
Nabogame
Puruandiro
Xochimilco
Tenango del Aire
San Pedro
Ixtlan
Allopatric
0
50
100
150
Mb
Chromosome 10: Maize
El Porvenir
Opopeo
Santa Clara
Nabogame
Puruandiro
Xochimilco
Tenango del Aire
San Pedro
Ixtlan
HAPMIX
STRUCTURE
0
50
100
150
Mb
