# Supplementary material for: The Genomic Signature of Crop-Wild Introgression in Maize
Source: PLoS Genet. 2013 May 9;9(5):e1003477. doi: 10.1371/journal.pgen.1003477 (PMC3649989; doi:10.1371/journal.pgen.1003477)
Supplement: Figure S8 — Identity by State (IBS) of modern maize lines with mexicana and parviglumis across each chromosome. All plots are as in Figure 5B. (PDF) [file pgen.1003477.s008.pdf]

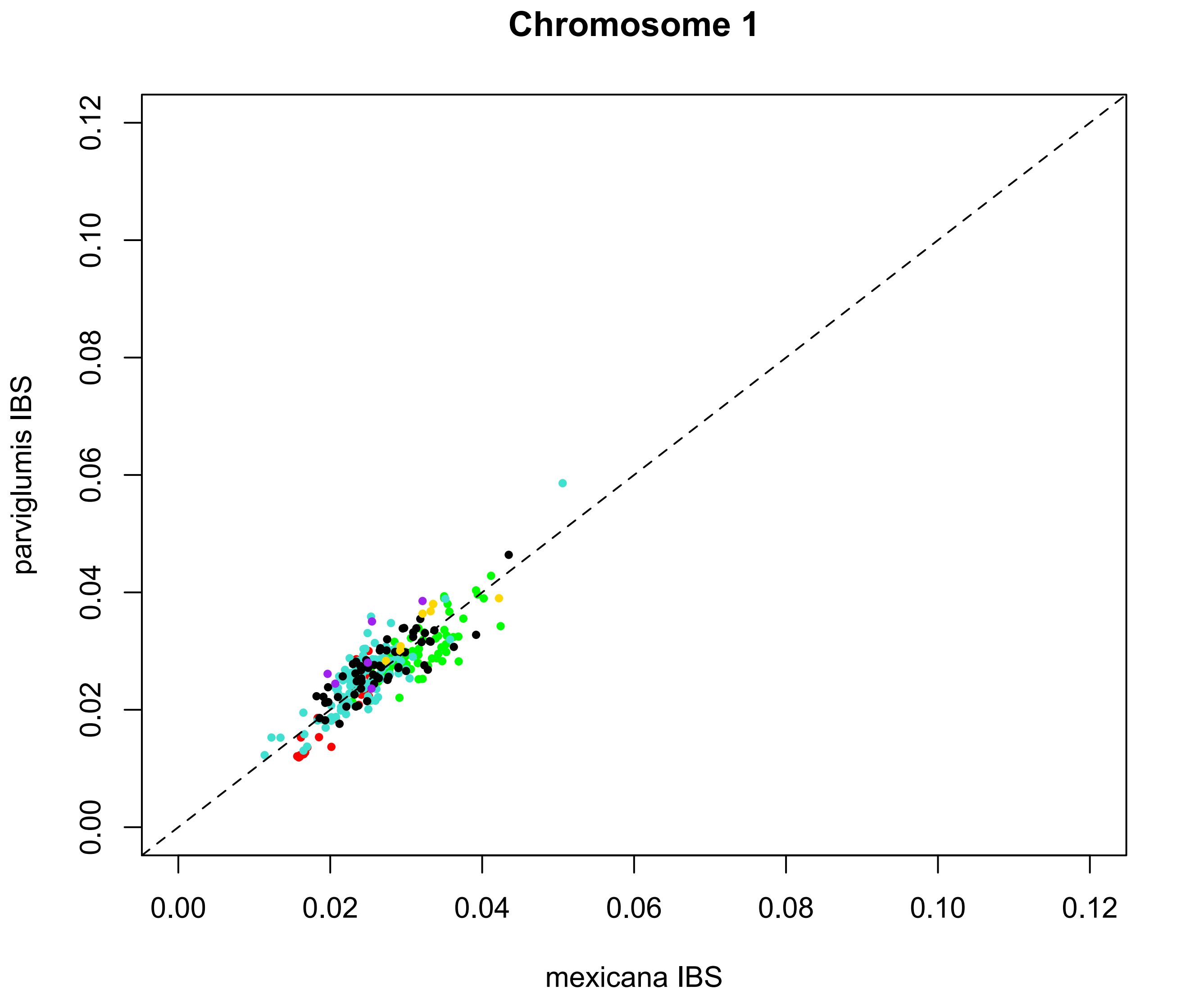
Chromosome 1
parviglumis IBS
mexicana IBS
0.00
0.02
0.04
0.06
0.08
0.10
0.12

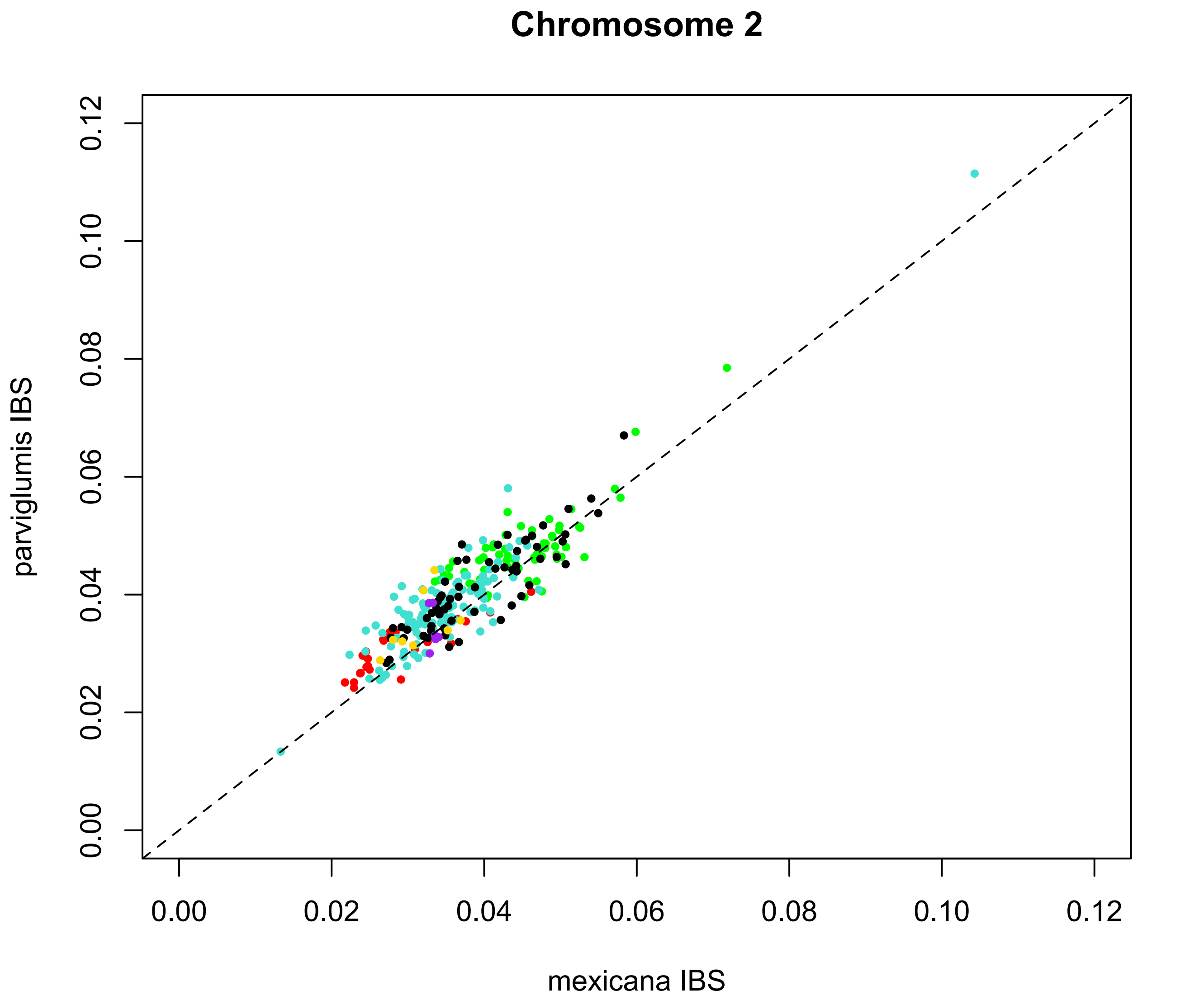
Chromosome 2
parviglumis IBS
mexicana IBS
0.00
0.02
0.04
0.06
0.08
0.10
0.12

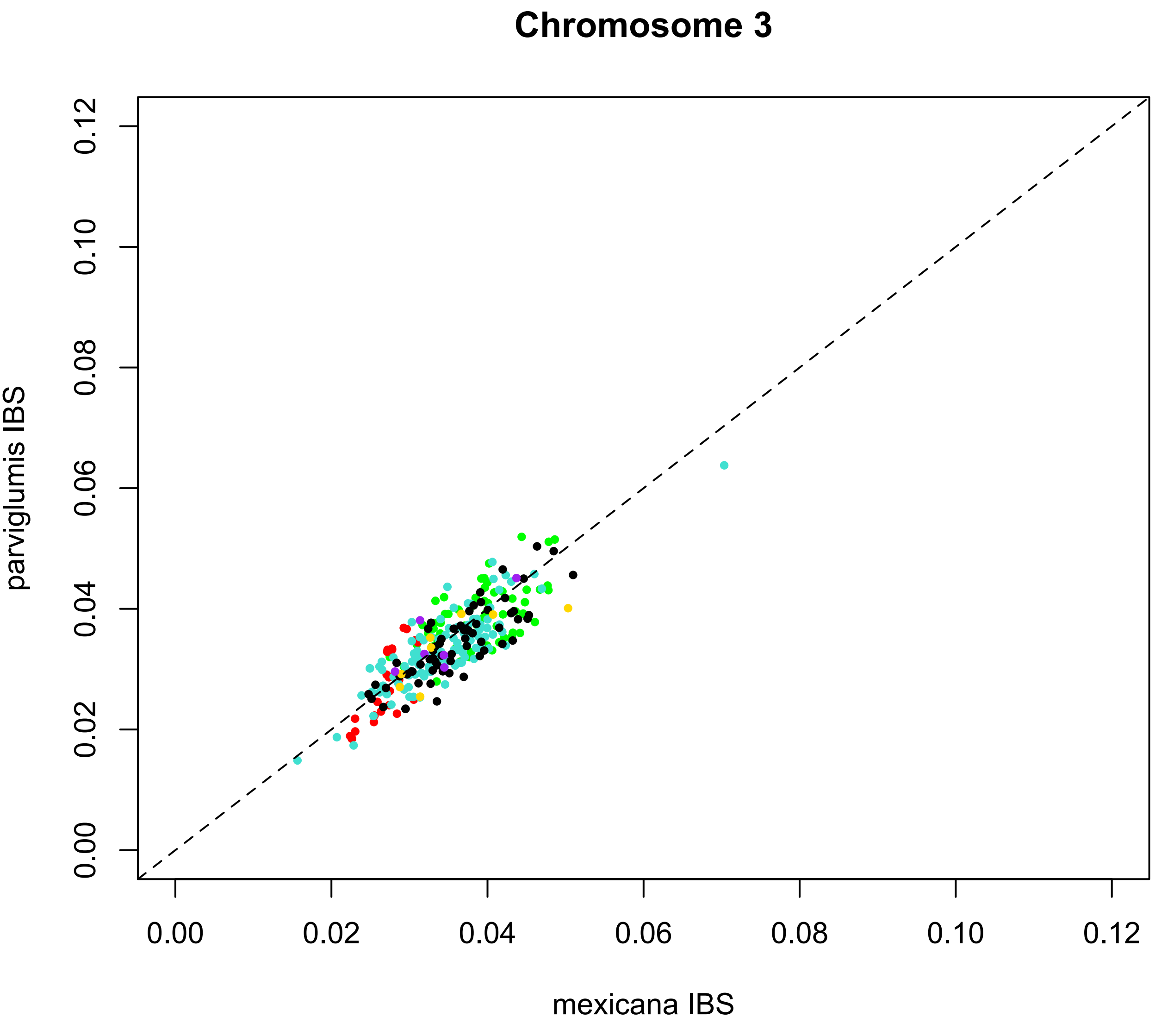

Chromosome 3
parviglumis IBS
mexicana IBS
0.00
0.02
0.04
0.06
0.08
0.10
0.12

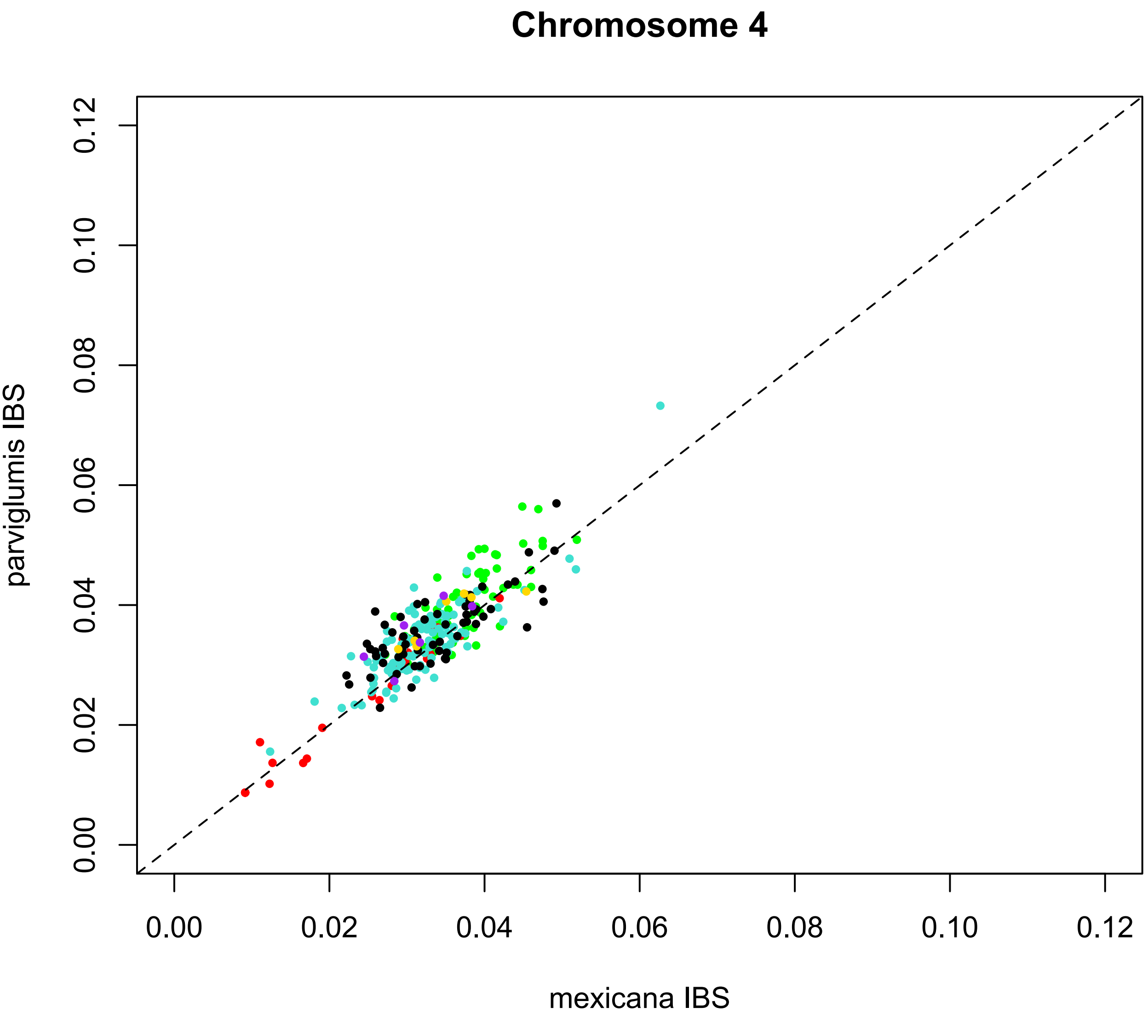

Chromosome 4
parviglumis IBS
mexicana IBS
0.00
0.02
0.04
0.06
0.08
0.10
0.12

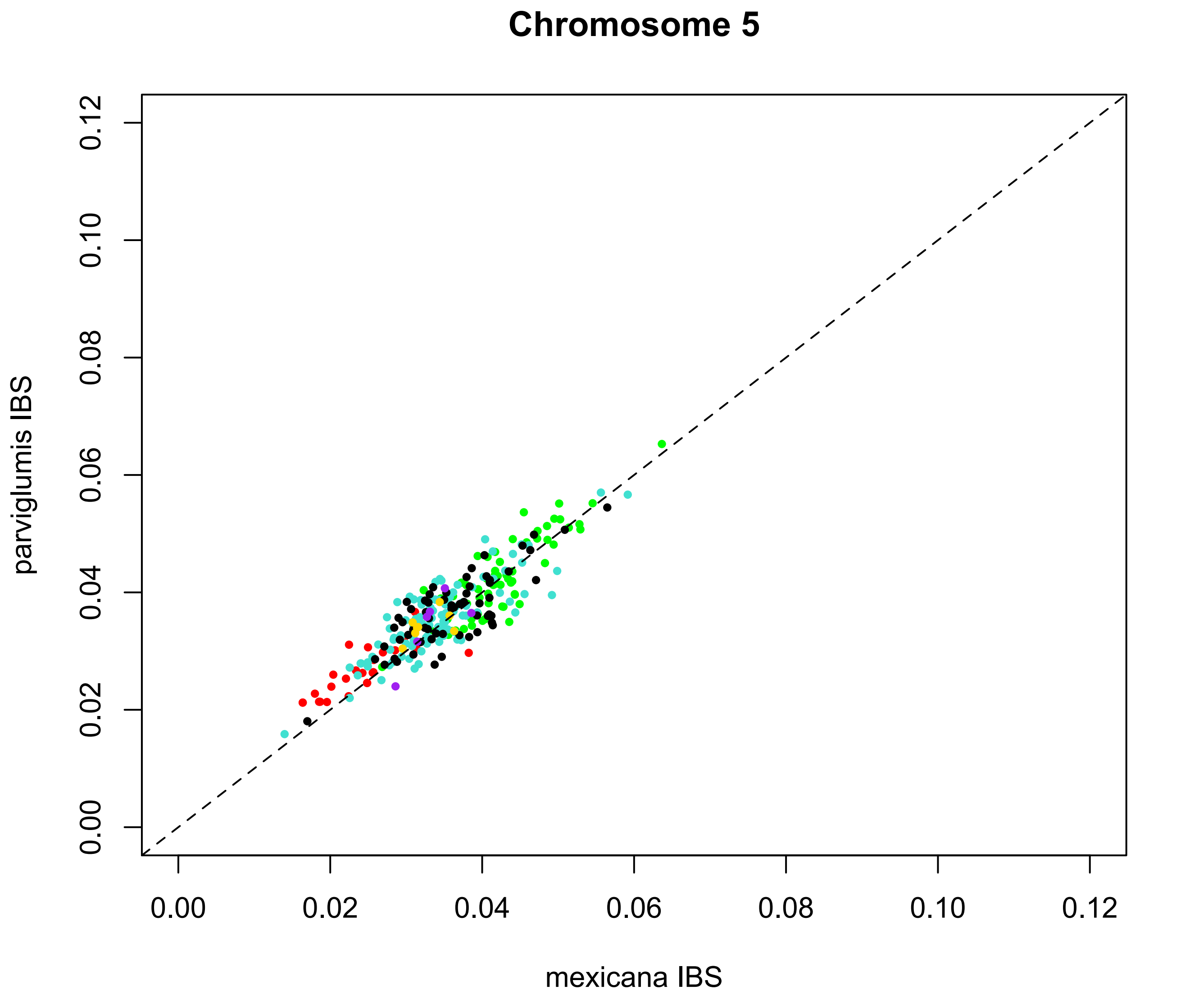
Chromosome 5
parviglumis IBS
mexicana IBS
0.00
0.02
0.04
0.06
0.08
0.10
0.12

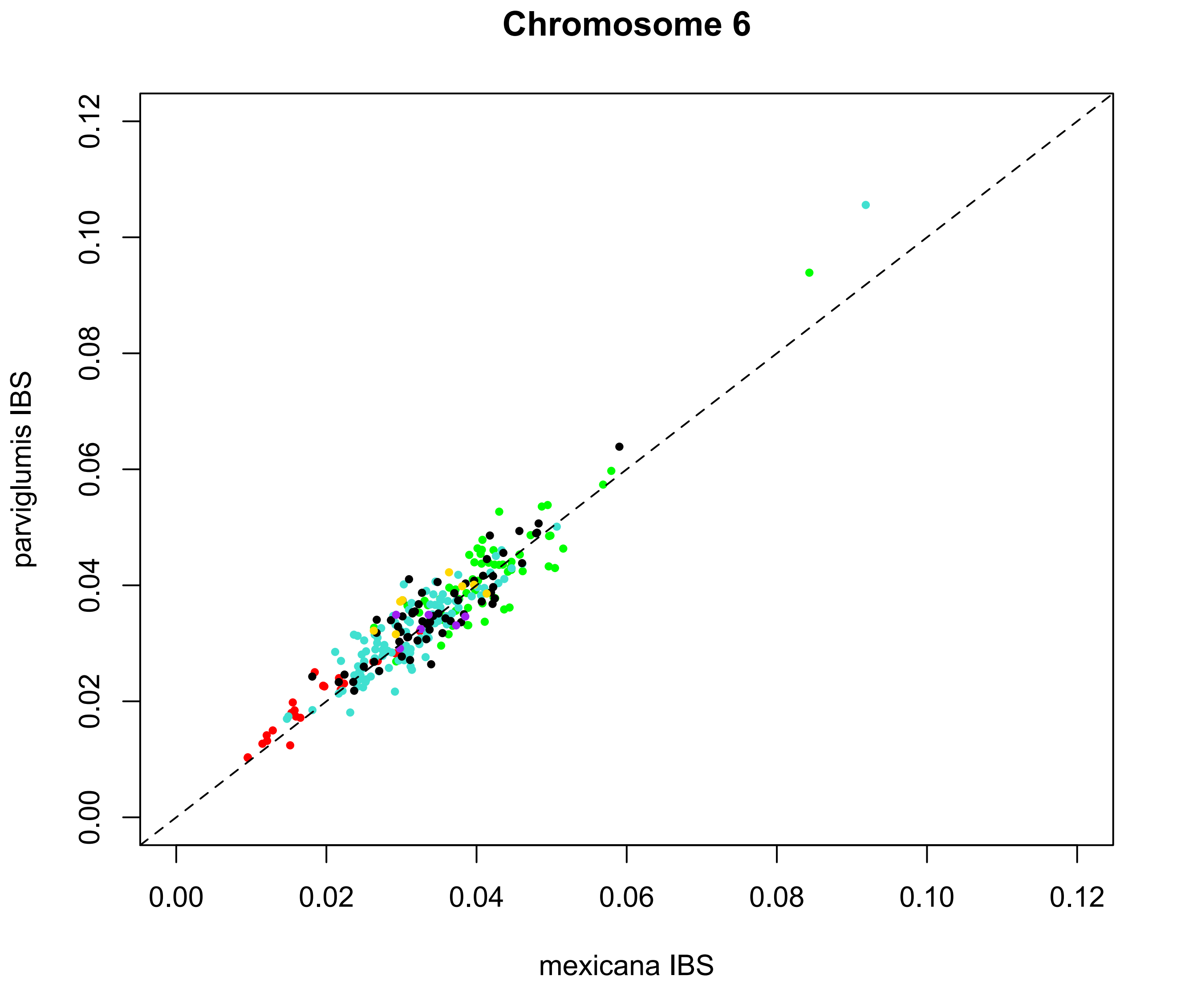
Chromosome 6
parviglumis IBS
mexicana IBS
0.00
0.02
0.04
0.06
0.08
0.10
0.12

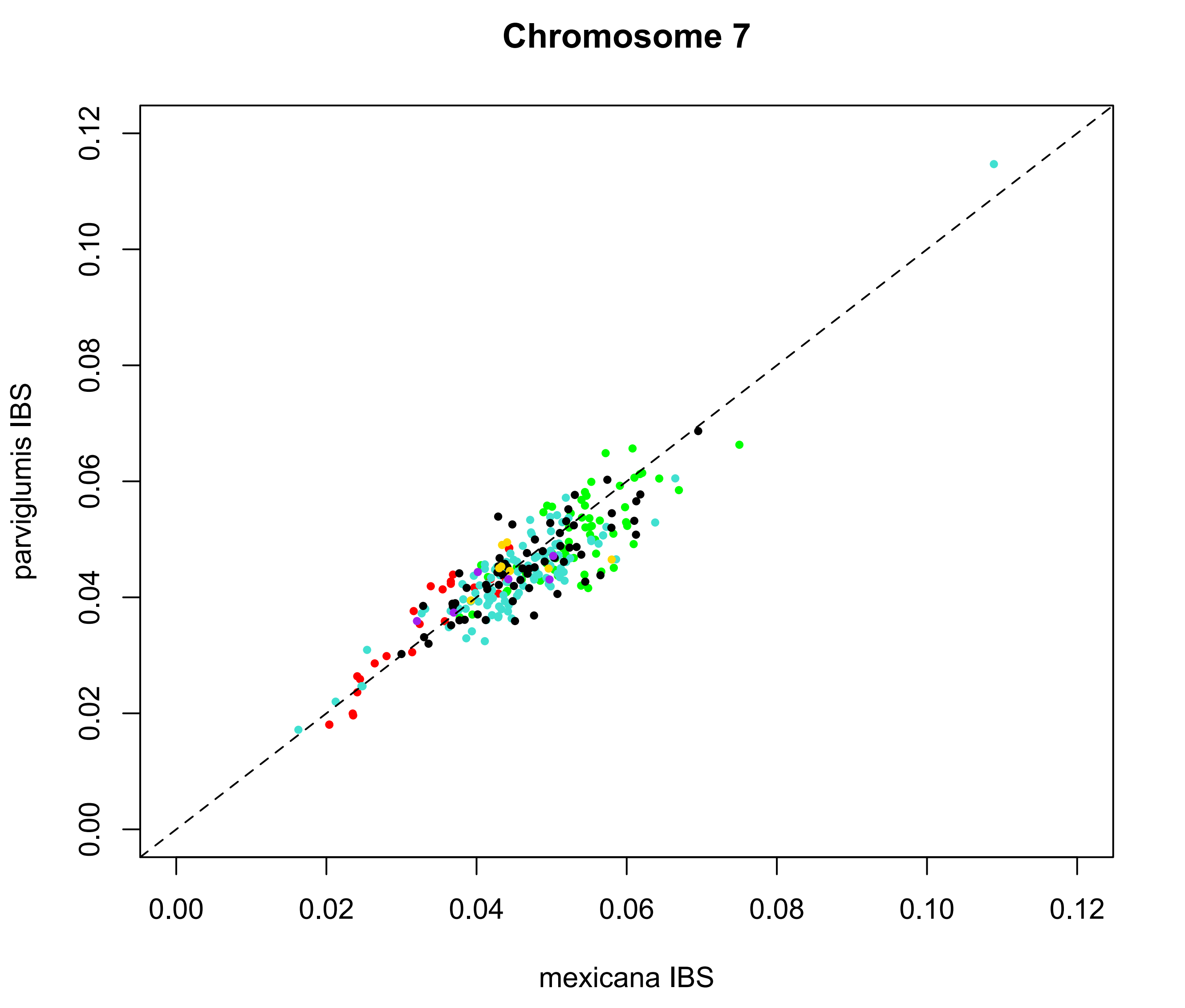
Chromosome 7
parviglumis IBS
mexicana IBS
0.00
0.02
0.04
0.06
0.08
0.10
0.12

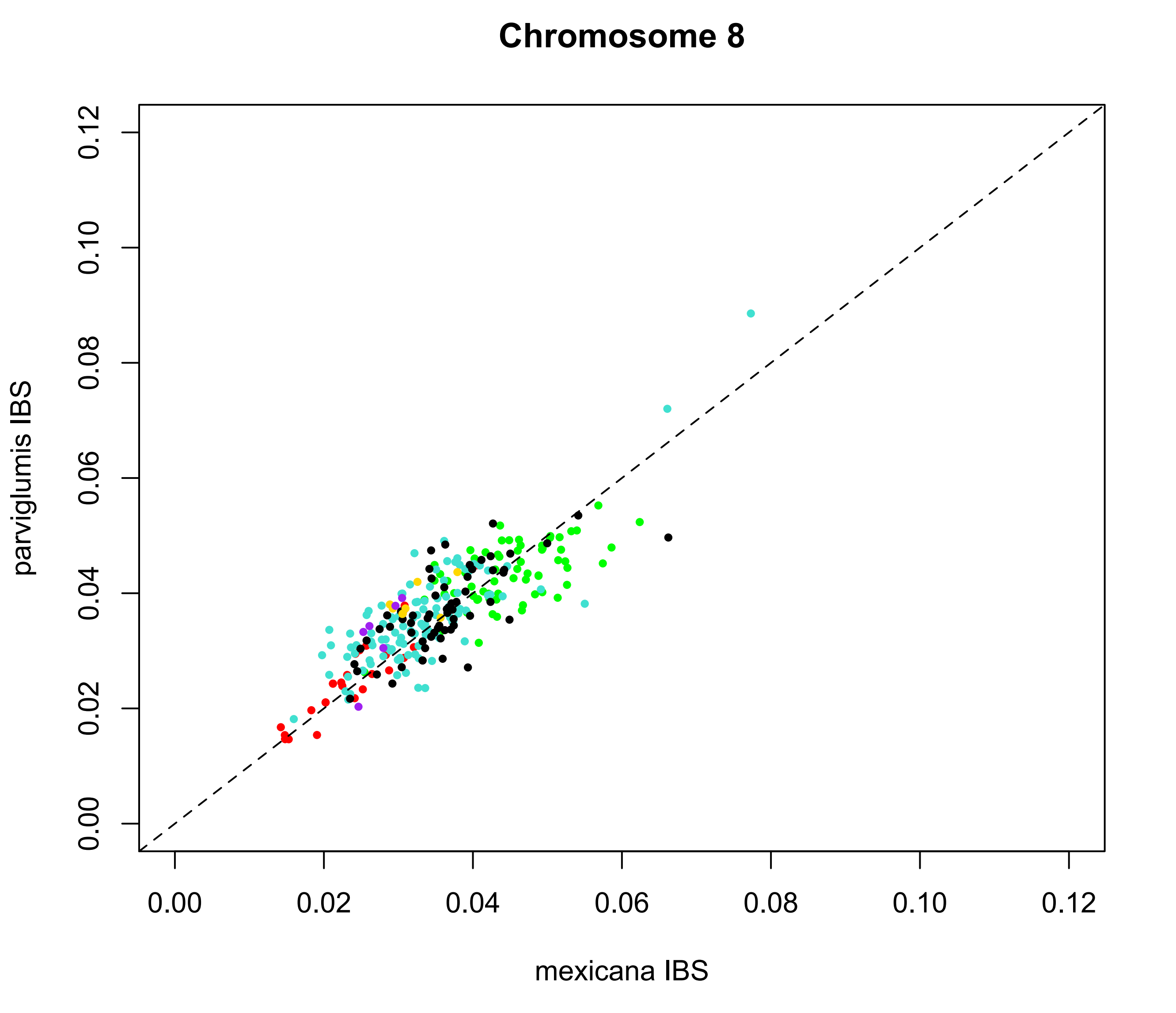
Chromosome 8
parviglumis IBS
mexicana IBS
0.00
0.02
0.04
0.06
0.08
0.10
0.12

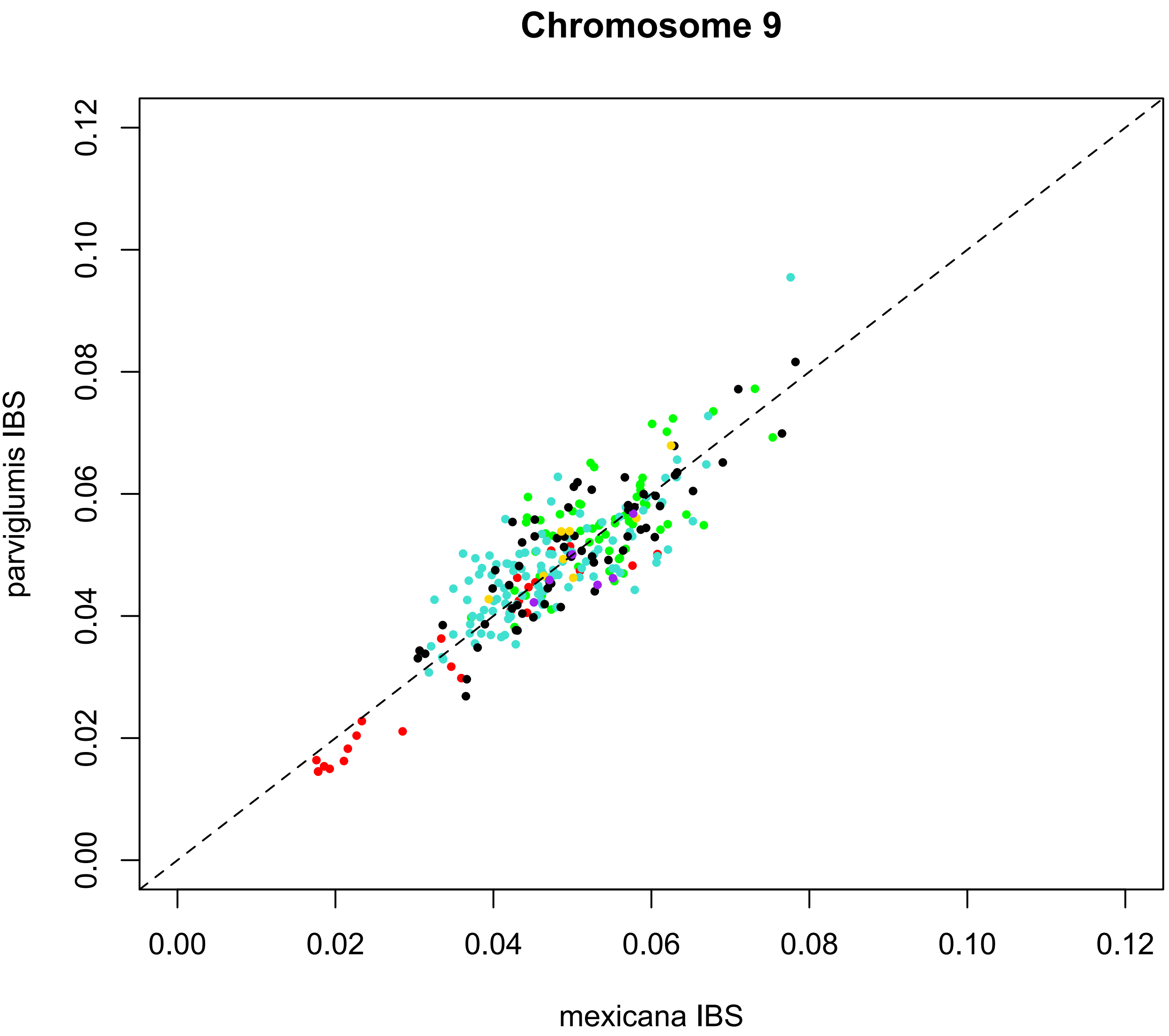

Chromosome 9
parviglumis IBS
mexicana IBS
0.00
0.02
0.04
0.06
0.08
0.10
0.12

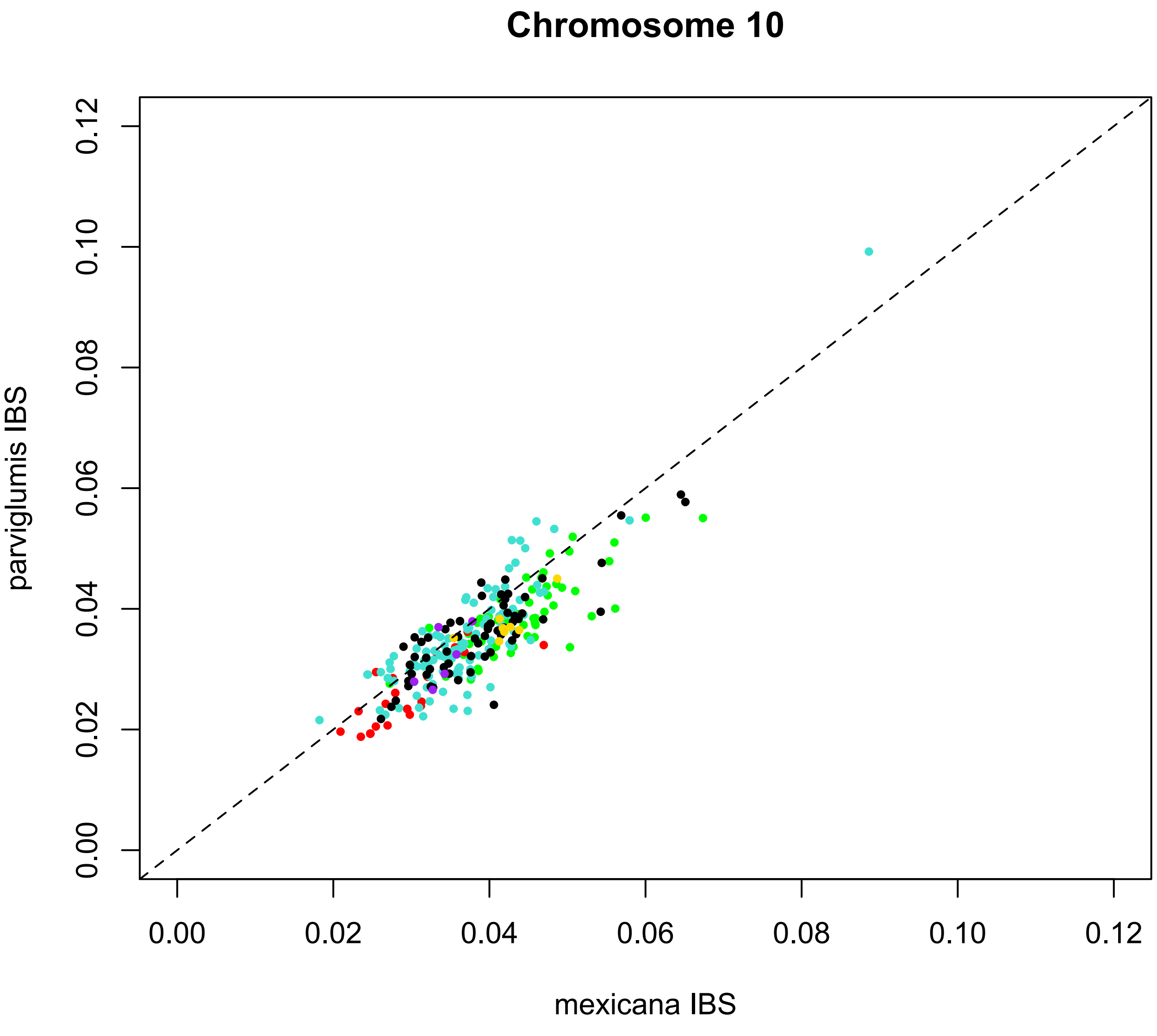
Chromosome 10
parviglumis IBS
mexicana IBS
0.00
0.02
0.04
0.06
0.08
0.10
0.12
