# Supplementary material for: The Genomic Signature of Crop-Wild Introgression in Maize
Source: PLoS Genet. 2013 May 9;9(5):e1003477. doi: 10.1371/journal.pgen.1003477 (PMC3649989; doi:10.1371/journal.pgen.1003477)
Supplement: Figure S9 — The difference between IBS modern maize/mexicana and IBS modern maize/parviglumis across each chromosome. All plots are as in Figure 5A. Dashed lines indicate genomic regions of mexicana introgression into highland Mexican maize conserved across ≥7 populations. (PDF) [file pgen.1003477.s009.pdf]

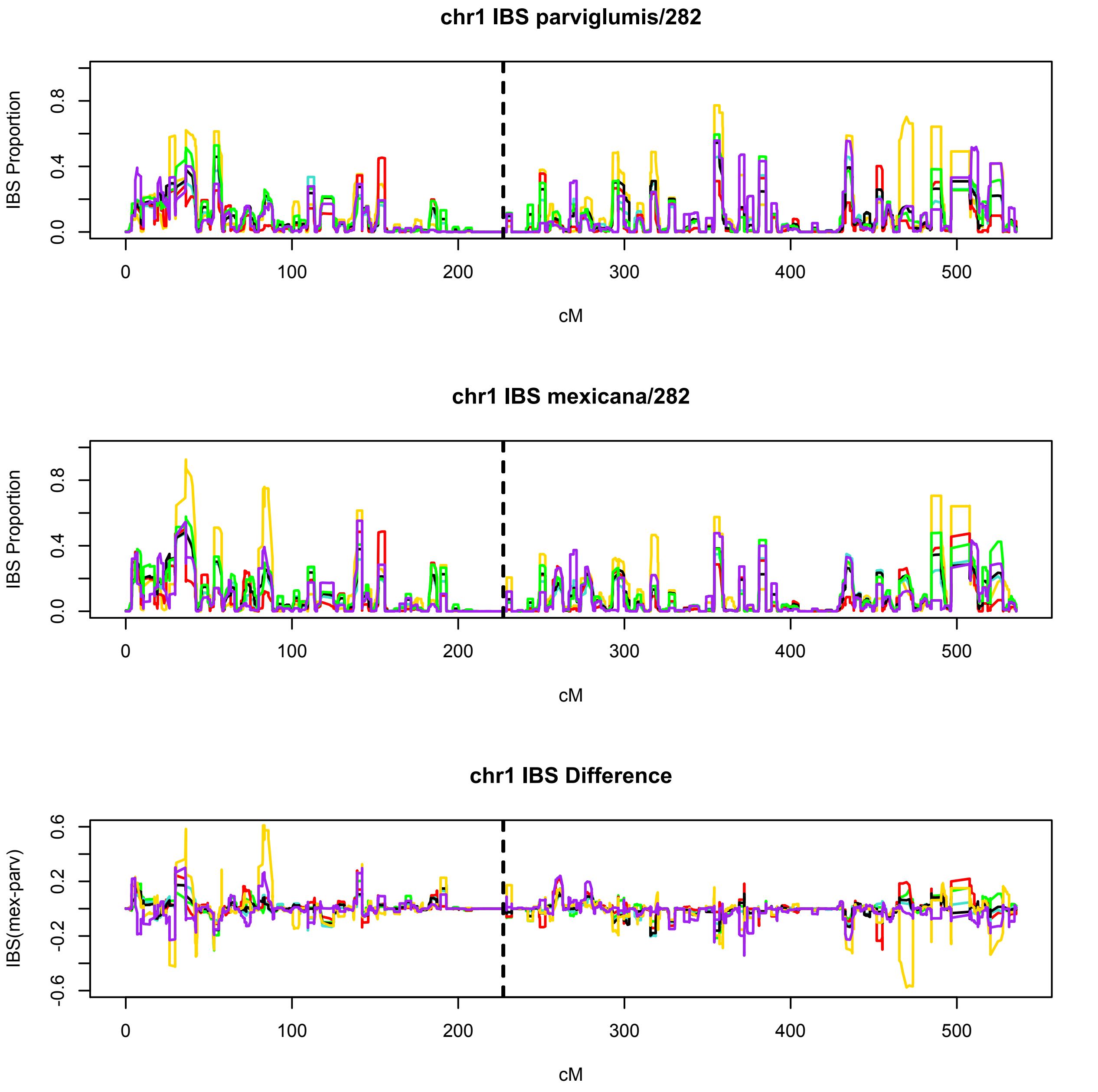

chr1 IBS parviglumis/282
IBS Proportion
0.0
0.4
0.8
0
100
200
300
400
500
cM
chr1 IBS mexicana/282
IBS Proportion
0.0
0.4
0.8
0
100
200
300
400
500
cM
chr1 IBS Difference
IBS(mex-parv)
-0.6
-0.2
0.2
0.6
0
100
200
300
400
500
cM

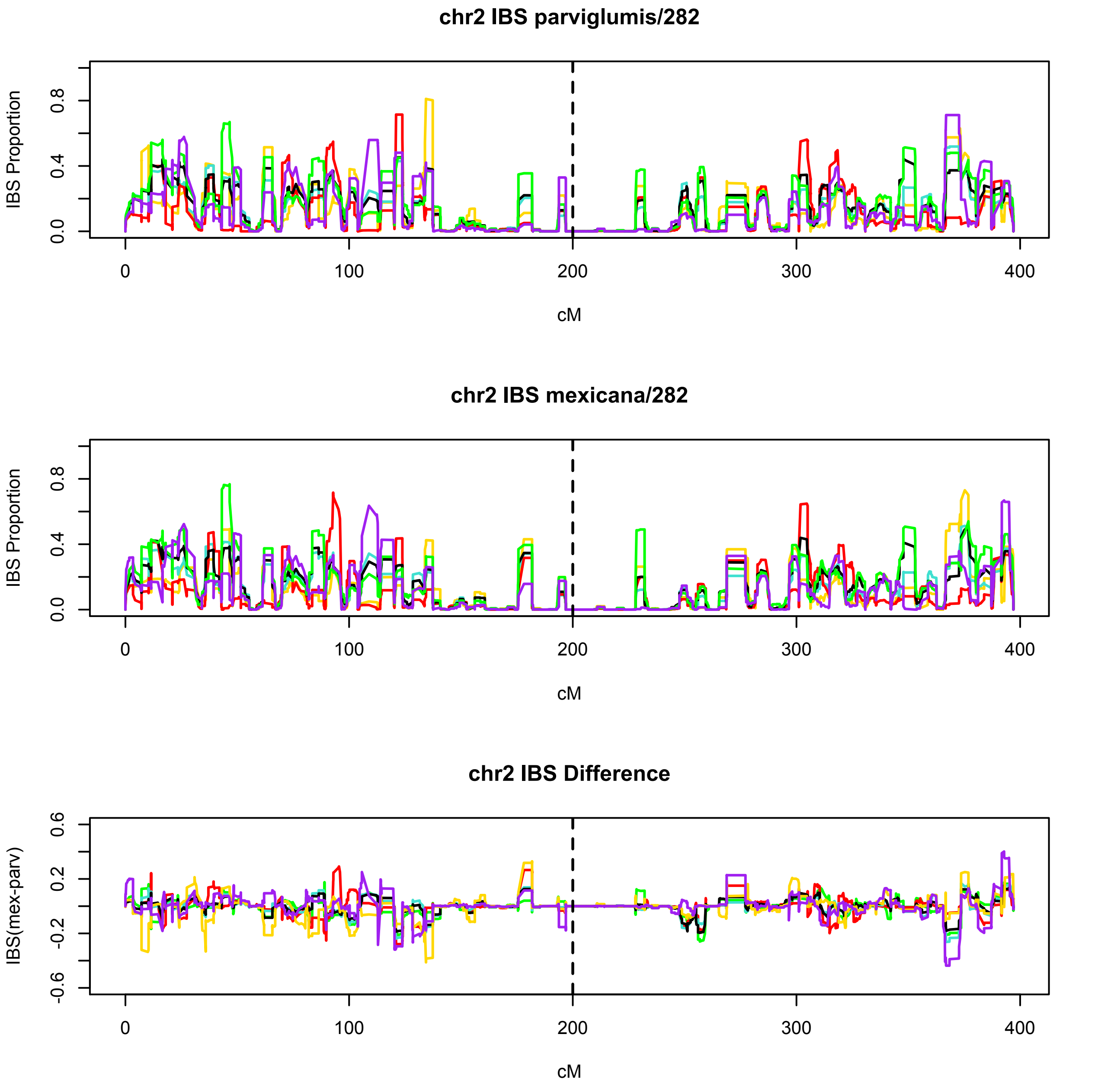
chr2 IBS parviglumis/282
IBS Proportion
0.0
0.4
0.8
0
100
200
300
400
cM
chr2 IBS mexicana/282
IBS Proportion
0.0
0.4
0.8
0
100
200
300
400
cM
chr2 IBS Difference
IBS(mex-parv)
-0.6
-0.2
0.2
0.6
0
100
200
300
400
cM

chr3 IBS parviglumis/282

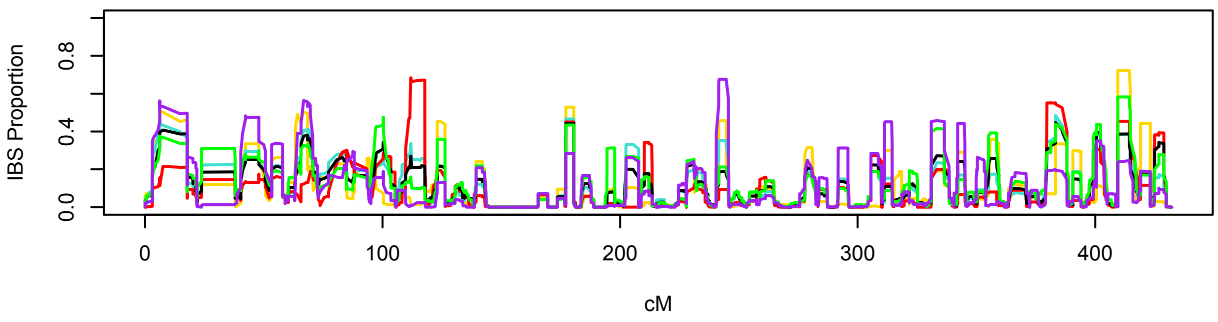

chr3 IBS mexicana/282

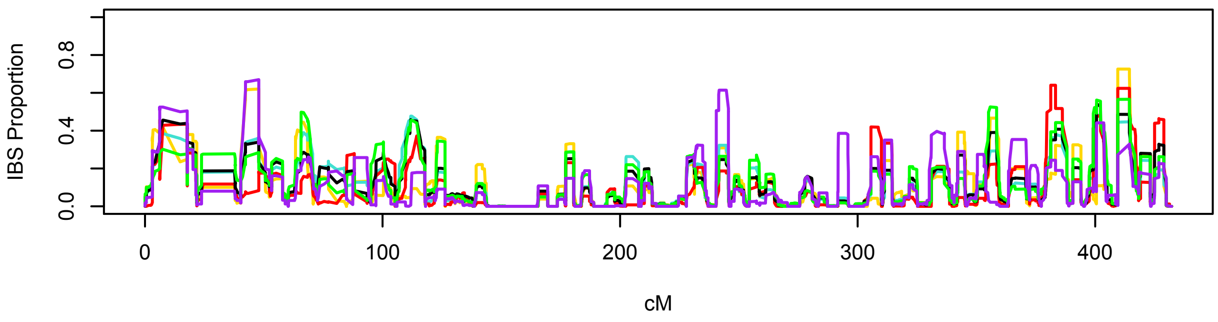

chr3 IBS Difference

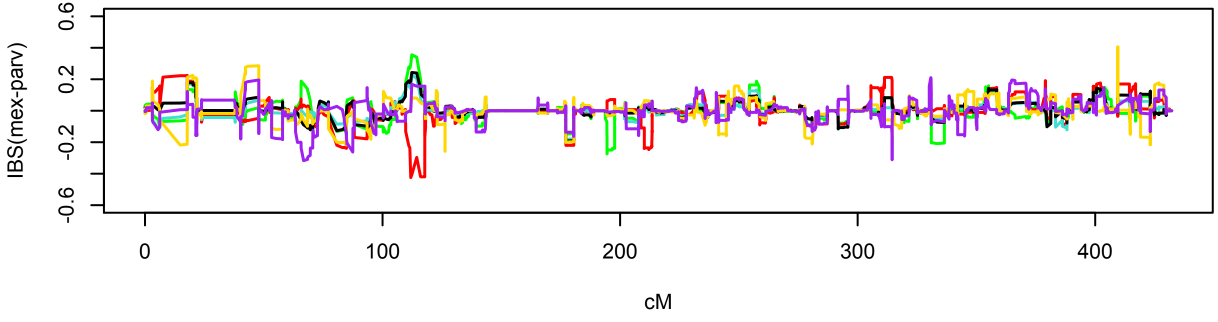

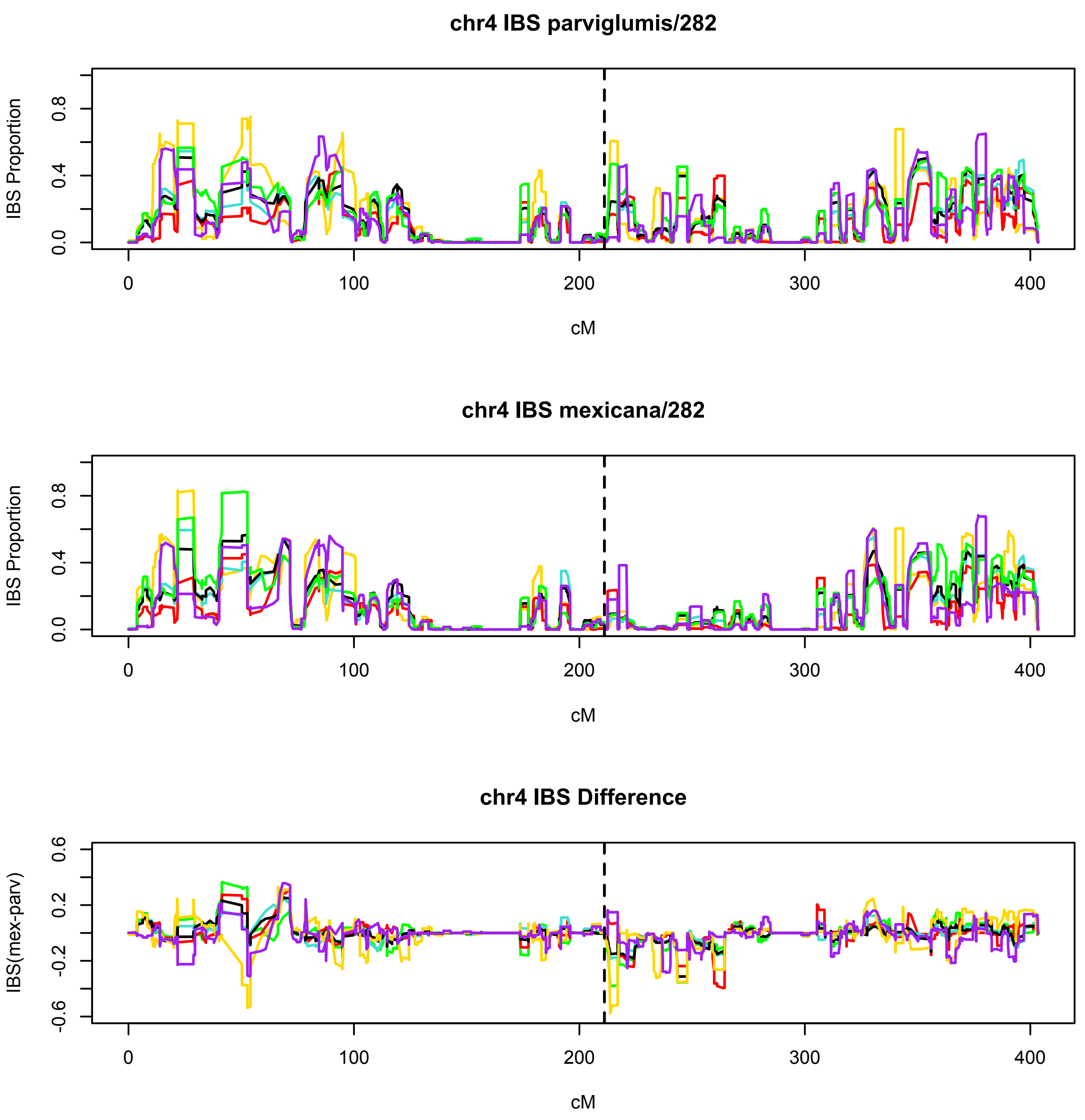
chr4 IBS parviglumis/282
IBS Proportion
0.0
0.4
0.8
0
100
200
300
400
cM
chr4 IBS mexicana/282
IBS Proportion
0.0
0.4
0.8
0
100
200
300
400
cM
chr4 IBS Difference
IBS(mex-parv)
-0.6
-0.2
0.2
0.6
0
100
200
300
400
cM

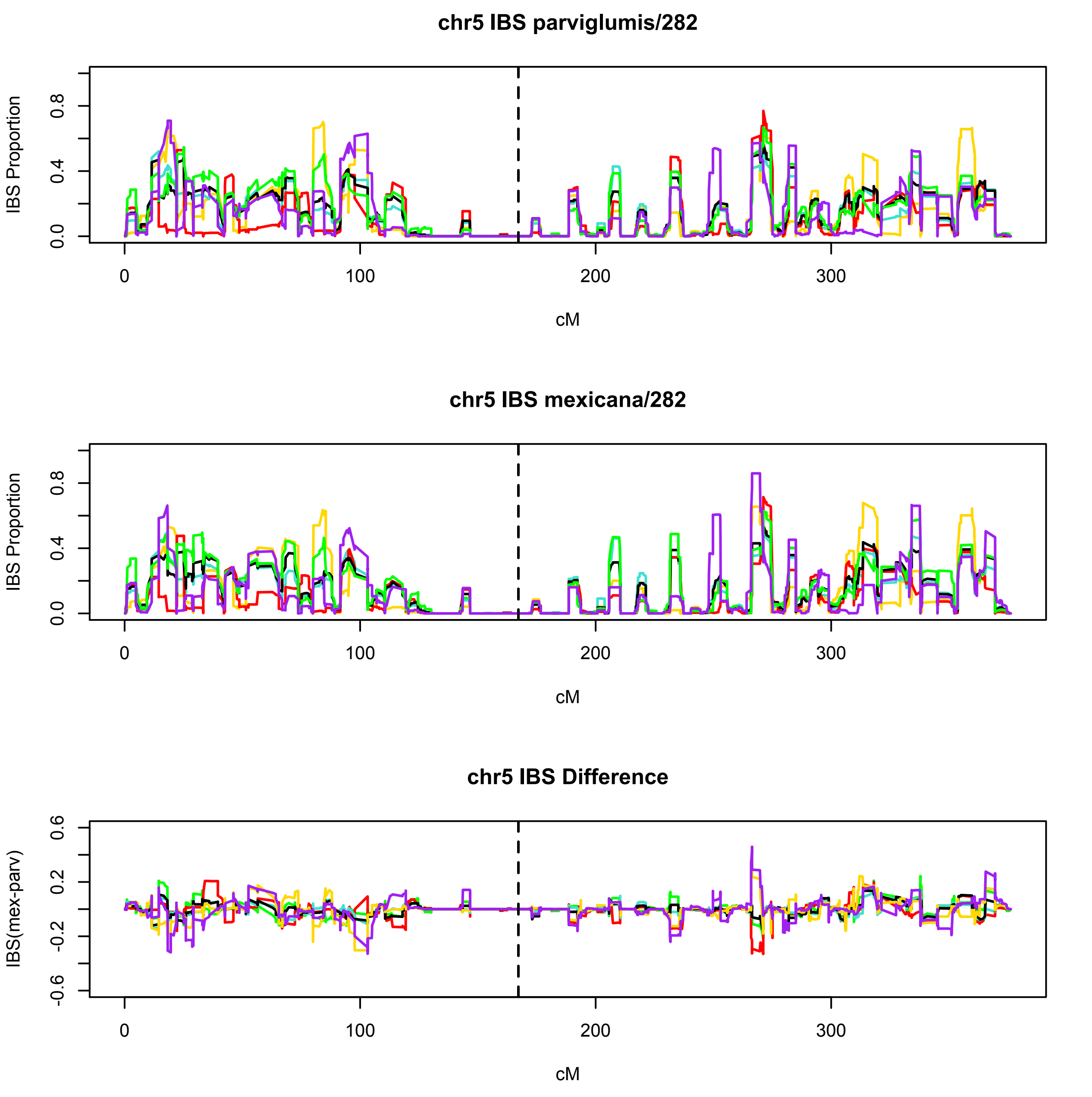
chr5 IBS parviglumis/282
IBS Proportion
0.0
0.4
0.8
0
100
200
300
cM
chr5 IBS mexicana/282
IBS Proportion
0.0
0.4
0.8
0
100
200
300
cM
chr5 IBS Difference
IBS(mex-parv)
-0.6
-0.2
0.2
0.6
0
100
200
300
cM

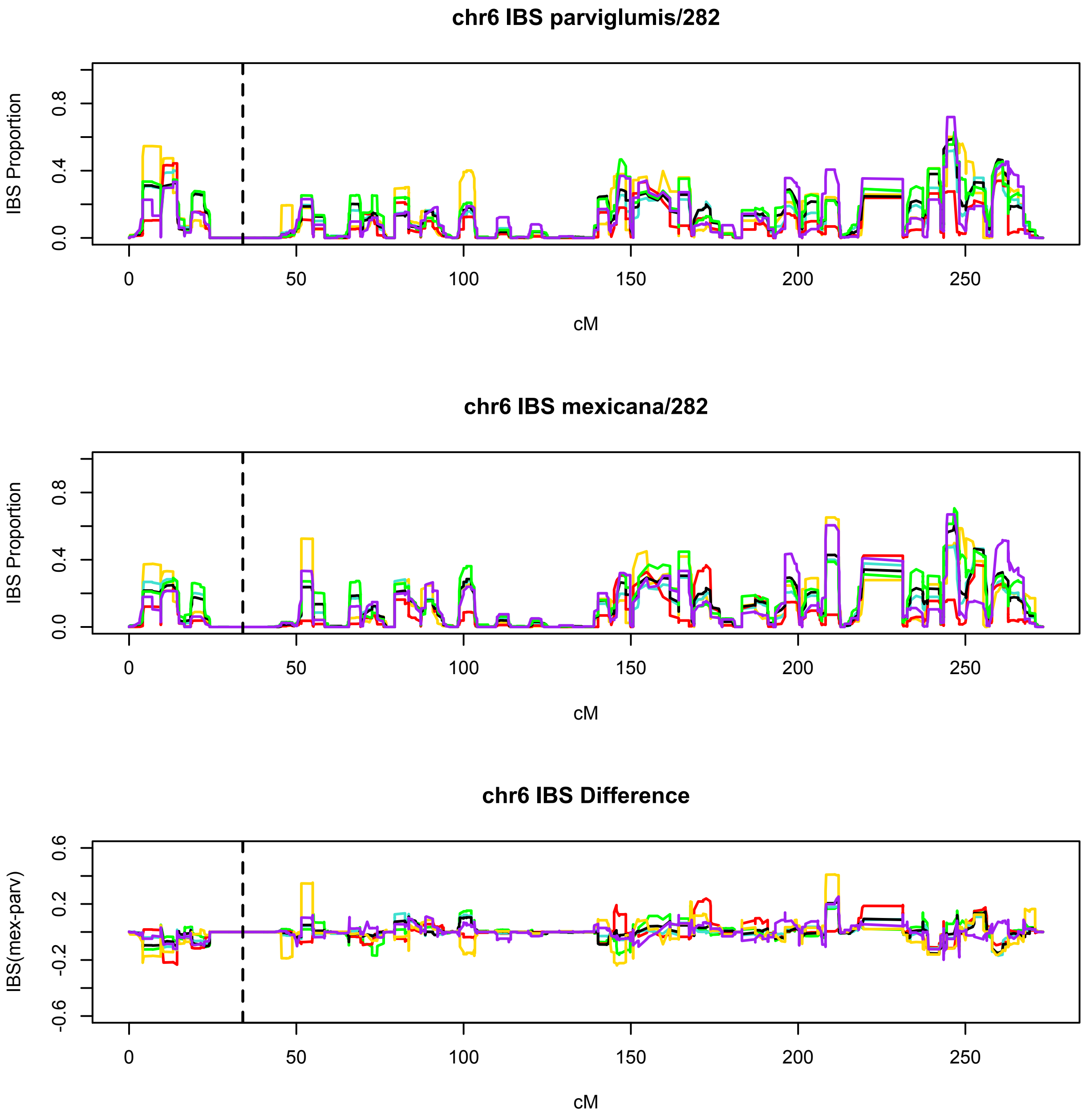

chr6 IBS parviglumis/282
IBS Proportion
0.0
0.4
0.8
0
50
100
150
200
250
cM
chr6 IBS mexicana/282
IBS Proportion
0.0
0.4
0.8
0
50
100
150
200
250
cM
chr6 IBS Difference
IBS(mex-parv)
-0.6
-0.2
0.2
0.6
0
50
100
150
200
250
cM

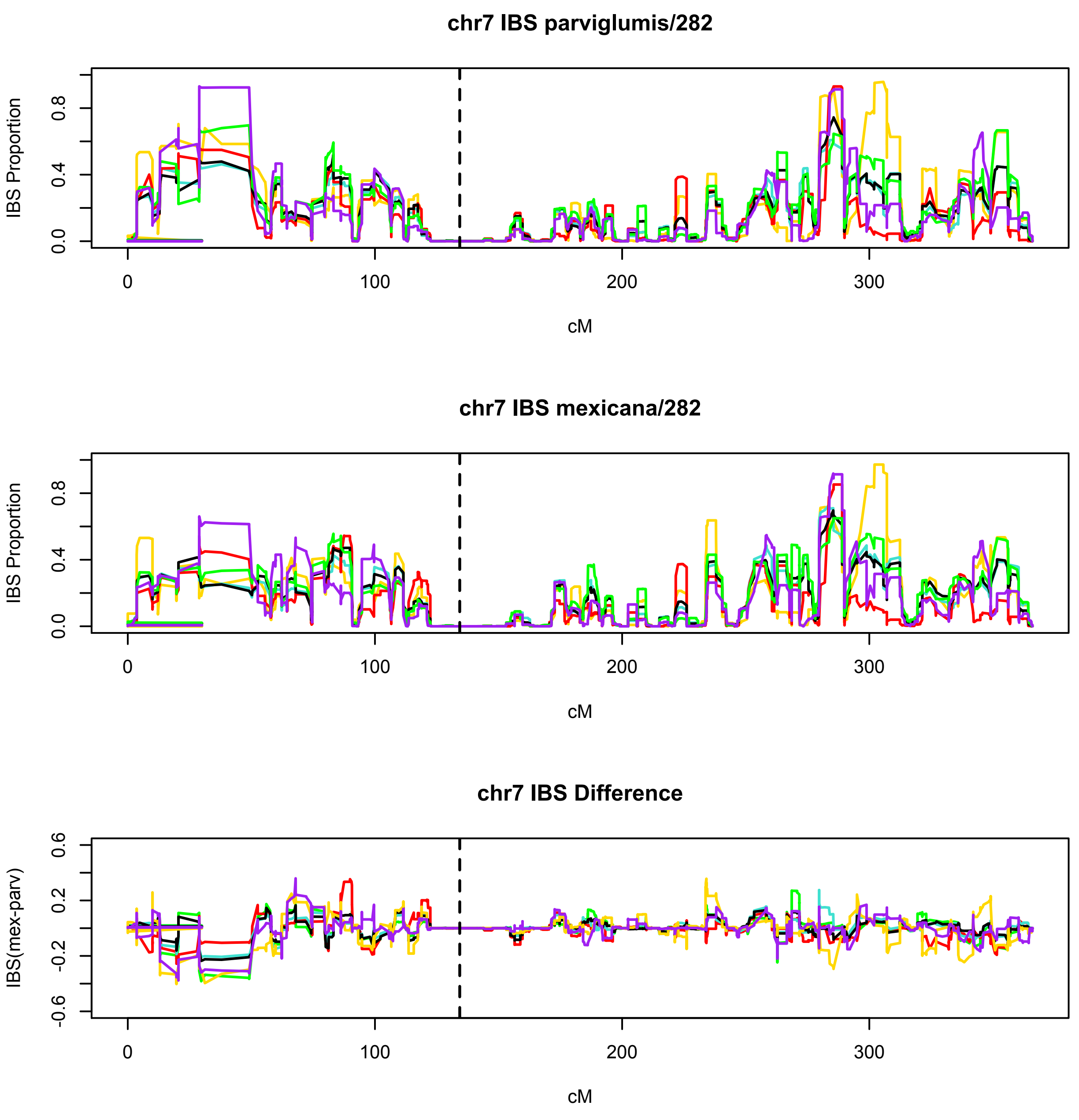
chr7 IBS parviglumis/282
IBS Proportion
0.0
0.4
0.8
0
100
200
300
cM
chr7 IBS mexicana/282
IBS Proportion
0.0
0.4
0.8
0
100
200
300
cM
chr7 IBS Difference
IBS(mex-parv)
-0.6
-0.2
0.2
0.6
0
100
200
300
cM

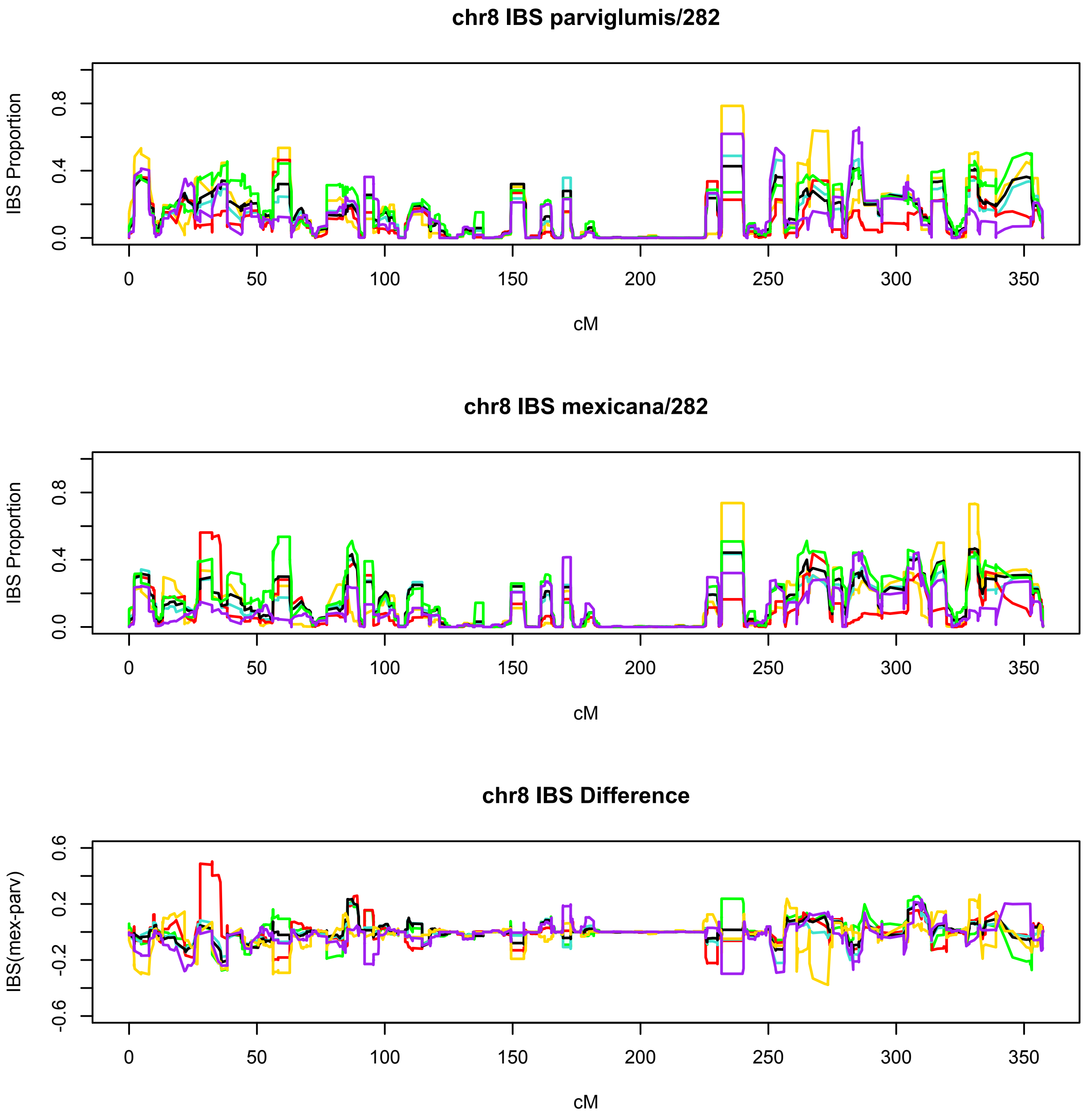
chr8 IBS parviglumis/282
IBS Proportion
0.0
0.4
0.8
0
50
100
150
200
250
300
350
cM
chr8 IBS mexicana/282
IBS Proportion
0.0
0.4
0.8
0
50
100
150
200
250
300
350
cM
chr8 IBS Difference
IBS(mex-parv)
-0.6
-0.2
0.2
0.6
0
50
100
150
200
250
300
350
cM

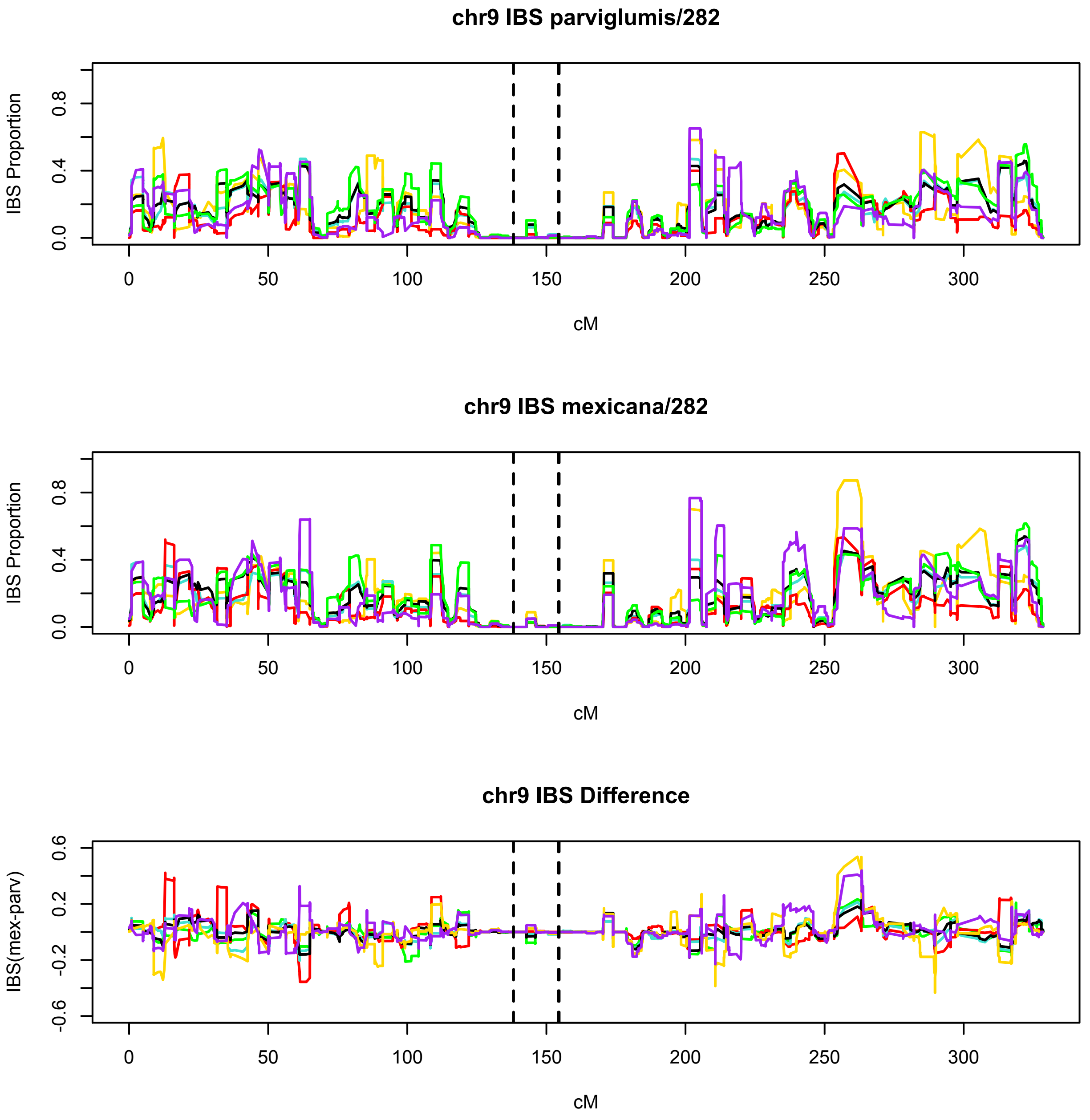
chr9 IBS parviglumis/282
IBS Proportion
0.0
0.4
0.8
0
50
100
150
200
250
300
cM
chr9 IBS mexicana/282
IBS Proportion
0.0
0.4
0.8
0
50
100
150
200
250
300
cM
chr9 IBS Difference
IBS(mex-parv)
-0.6
-0.2
0.2
0.6
0
50
100
150
200
250
300
cM

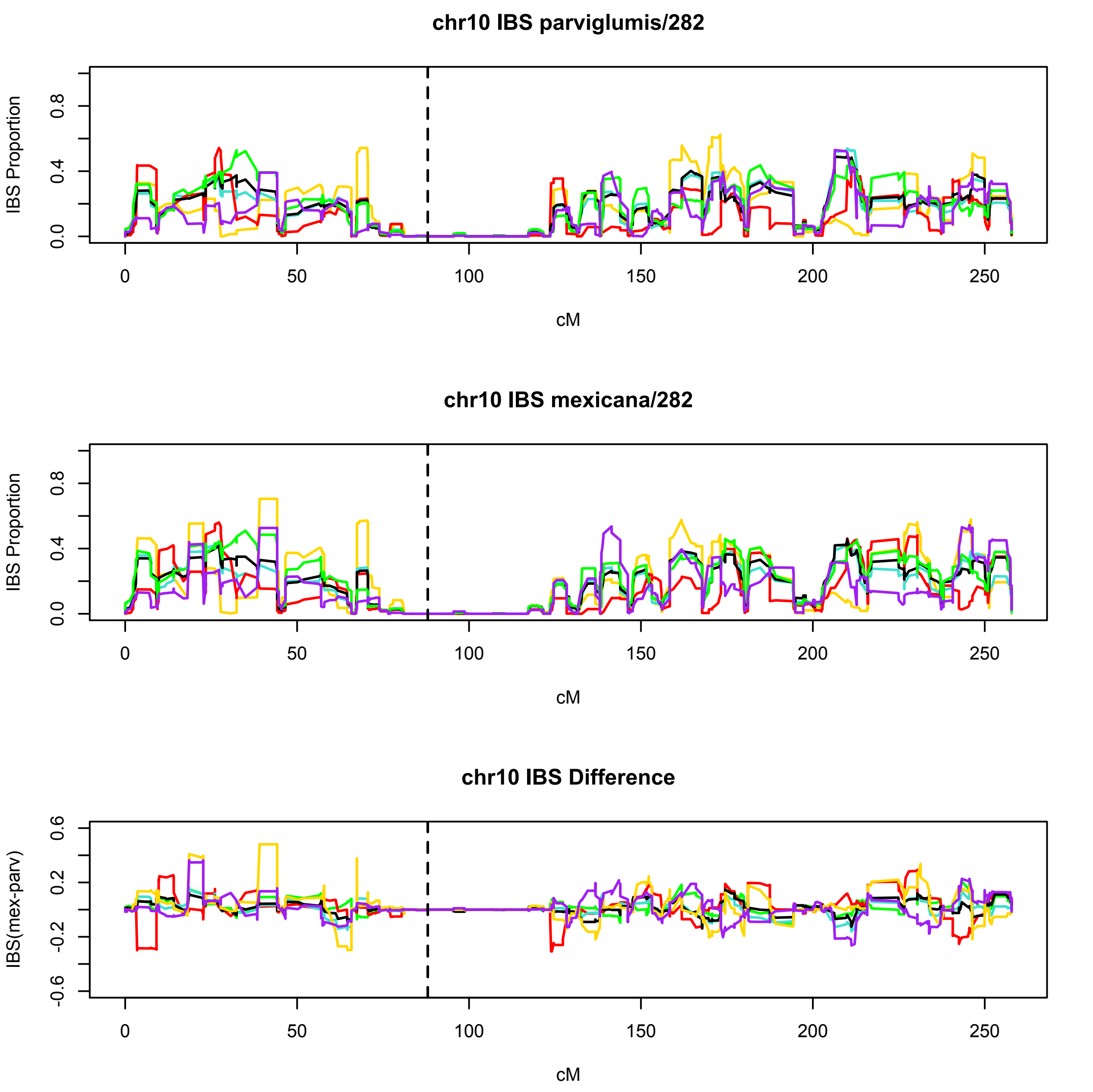
chr10 IBS parviglumis/282
IBS Proportion
0.0
0.4
0.8
0
50
100
150
200
250
cM
chr10 IBS mexicana/282
IBS Proportion
0.0
0.4
0.8
0
50
100
150
200
250
cM
chr10 IBS Difference
IBS(mex-parv)
-0.6
-0.2
0.2
0.6
0
50
100
150
200
250
cM
