# Supplementary material for: The Genomic Signature of Crop-Wild Introgression in Maize
Source: PLoS Genet. 2013 May 9;9(5):e1003477. doi: 10.1371/journal.pgen.1003477 (PMC3649989; doi:10.1371/journal.pgen.1003477)
Supplement: Table S1 — Sampling information for mexicana and maize populations. (PDF) [file pgen.1003477.s010.pdf]

Sampling Locality	State	Latitude	Longitude	Elevation	Comments
El Porvenir	Michoacan	19.68	-100.64	2094	Sympatric site
Ixtlan	Michoacan	20.17	-102.37	1547	Sympatric site
Nabogame	Chihuahua	26.25	-106.92	2020	Sympatric site
Opopeo	Michoacan	19.42	-101.61	2213	Sympatric site
Puruandiro	Michoacan	20.11	-101.49	1915	Sympatric site
San Pedro	Puebla	19.09	-98.49	2459	Sympatric site
Santa Clara	Michoacan	19.42	-101.64	2173	Sympatric site
Tenango del Aire	Mexico	19.12	-99.59	2609	Sympatric site
Xochimilco	Federal District	19.29	-99.08	2237	Sympatric site
Amatlan	Morelos	18.97	-99.03	1658	Allopatric *mexicana*
