# Supplementary material for: The Genomic Signature of Crop-Wild Introgression in Maize
Source: PLoS Genet. 2013 May 9;9(5):e1003477. doi: 10.1371/journal.pgen.1003477 (PMC3649989; doi:10.1371/journal.pgen.1003477)
Supplement: Table S2 — Summaries of diversity across mexicana and maize populations. HE = expected heterozygosity, %P = percent polymorphic loci, HO = observed heterozygosity, FIS = inbreeding coefficient calculated as (HE−HO)/HE. (PDF) [file pgen.1003477.s011.pdf]

Sampling Locality	H_E Maize	H_E *mexicana*	%*P* Maize	%*P* *mexicana*	H_O Maize	H_O *mexicana*	F_{IS} Maize	F_{IS} *mexicana*
El Porvenir	0.308	0.215	0.837	0.704	0.307	0.199	0.004	0.073
Ixtlan	0.224	0.202	0.515	0.668	0.210	0.172	0.063	0.148
Nabogame	0.307	0.185	0.830	0.675	0.299	0.171	0.025	0.078
Opopeo	0.296	0.212	0.810	0.679	0.287	0.204	0.031	0.040
Puruandiro	0.328	0.248	0.875	0.785	0.318	0.231	0.032	0.069
San Pedro	0.303	0.198	0.808	0.612	0.297	0.190	0.021	0.042
Santa Clara	0.298	0.175	0.810	0.559	0.294	0.163	0.014	0.070
Tenango del Aire	0.277	0.201	0.763	0.653	0.276	0.185	0.005	0.078
Xochimilco	0.288	0.150	0.749	0.439	0.261	0.146	0.095	0.030
Puerta Encantada	XX	0.174	XX	0.517	XX	0.166	XX	0.047
