# Supplementary material for: The Genomic Signature of Crop-Wild Introgression in Maize
Source: PLoS Genet. 2013 May 9;9(5):e1003477. doi: 10.1371/journal.pgen.1003477 (PMC3649989; doi:10.1371/journal.pgen.1003477)
Supplement: Table S4 — Genomic coordinates of shared introgression regions. (PDF) [file pgen.1003477.s013.pdf]

chr	region of introgression	in Lauter QTL?	window 1 start	window 1 end	window 2 start	window 2 end
1	120-145Mb	no	120904094	121469572	144425995	145312340
2	73-78Mb	yes	73807029	78500359	NA	NA
4	169-180Mb	yes	168753601	169803287	NA	NA
5	102-135Mb	yes	102877443	113778281	133333397	135180623
6	46-56Mb	no	46111110	55854813	NA	NA
7	30-31Mb	no	30664165	31314057	NA	NA
9	107-125Mb	yes	107315114	107840288	NA	NA
9	43Mb	yes	43903996	43903996	43287718	43287718
10	39-54Mb	yes	39590245	53484238	NA	NA
